# A Gammaherpesvirus Bcl-2 Ortholog Blocks B Cell Receptor-Mediated Apoptosis and Promotes the Survival of Developing B Cells *In Vivo*

**DOI:** 10.1371/journal.ppat.1003916

**Published:** 2014-02-06

**Authors:** Carrie B. Coleman, Jennifer E. McGraw, Emily R. Feldman, Alexa N. Roth, Lisa R. Keyes, Katrina R. Grau, Stephanie L. Cochran, Thomas J. Waldschmidt, Chengyu Liang, J. Craig Forrest, Scott A. Tibbetts

**Affiliations:** 1 Department of Molecular Genetics & Microbiology and UF Shands Cancer Center, College of Medicine, University of Florida, Gainesville, Florida, United States of America; 2 Department of Pathology, Carver College of Medicine, University of Iowa, Iowa City, Iowa, United States of America; 3 Department of Molecular Microbiology & Immunology, Keck School of Medicine, University of Southern California, Los Angeles, California, United States of America; 4 Department of Microbiology & Immunology, University of Arkansas for Medical Sciences, Little Rock, Arkansas, United States of America; University of Alberta, Canada

## Abstract

Gammaherpesviruses such as Epstein-Barr virus (EBV) and Kaposi's sarcoma-associated herpesvirus (KSHV, HHV-8) establish lifelong latency in their hosts and are associated with the development of several types of malignancies, including a subset of B cell lymphomas. These viruses are thought to co-opt the process of B cell differentiation to latently infect a fraction of circulating memory B cells, resulting in the establishment of a stable latency setpoint. However, little is known about how this infected memory B cell compartment is maintained throughout the life of the host. We have previously demonstrated that immature and transitional B cells are long-term latency reservoirs for murine gammaherpesvirus 68 (MHV68), suggesting that infection of developing B cells contributes to the maintenance of lifelong latency. During hematopoiesis, immature and transitional B cells are subject to B cell receptor (BCR)-mediated negative selection, which results in the clonal deletion of autoreactive B cells. Interestingly, numerous gammaherpesviruses encode homologs of the anti-apoptotic protein Bcl-2, suggesting that virus inhibition of apoptosis could subvert clonal deletion. To test this, we quantified latency establishment in mice inoculated with MHV68 vBcl-2 mutants. vBcl-2 mutant viruses displayed a marked decrease in the frequency of immature and transitional B cells harboring viral genome, but this attenuation could be rescued by increased host Bcl-2 expression. Conversely, vBcl-2 mutant virus latency in early B cells and mature B cells, which are not targets of negative selection, was remarkably similar to wild-type virus. Finally, *in vivo* depletion of developing B cells during chronic infection resulted in decreased mature B cell latency, demonstrating a key role for developing B cells in the maintenance of lifelong latency. Collectively, these findings support a model in which gammaherpesvirus latency in circulating mature B cells is sustained in part through the recurrent infection and vBcl-2-mediated survival of developing B cells.

## Author Summary

Gammaherpesviruses such as Epstein-Barr virus and Kaposi's sarcoma herpesvirus are widespread pathogens that establish lifelong infections in a dormant state termed latency. Although most gammaherpesvirus infections are asymptomatic, infection of some individuals leads to the development of B cell lymphoma or other cancers. It is well known that during latency these viruses reside in mature B cells of the immune system; however, little is known about how this reservoir is maintained for life. Using murine gammaherpesvirus 68 infection of mice as a model to study gammaherpesvirus infections inside a living host, we have previously demonstrated that gammaherpesviruses can infect early precursors of B cells. In normal situations, the differentiation of such precursors into mature B cells is a tightly regulated process that leads to the death of cells that react inappropriately to host tissues. Here though, we demonstrate that a gammaherpesvirus protein called vBcl-2 can block the death of infected precursor B cells, and that vBcl-2 is critical for infection of these cells. Finally, we show that depleting precursor B cells reduces mature B cell latency. Together, these data suggest that vBcl-2 proteins play a key role in lifelong gammaherpesvirus latency and may be a potent target for future drug development.

## Introduction

The human gammaherpesviruses, Epstein-Barr virus (EBV) and Kaposi's sarcoma-associated herpesvirus (KSHV, HHV-8), and the genetically- and pathogenically-related murine gammaherpesvirus 68 (MHV68, γHV68, MuHV-4), establish lifelong latent infections in circulating B cells. B cells are a crucial component of the adaptive immune response as they are capable of mounting responses to an enormous range of antigens through the production of antibodies and the establishment of immunological memory. Hence, maintaining a fully functional and diverse B cell population is critical for protection against a variety of bacterial and viral infections. Although gammaherpesvirus infections have been linked with the development of a considerable number of malignancies including B cell lymphomas and Kaposi's sarcoma, such pathogenic outcomes occur rarely in healthy hosts and have vastly increased prevalence in immunosuppressed populations [1]–[3]. Thus, gammaherpesviruses have evolved a symbiotic relationship with the host immune system in which they are able to maintain lifelong infection in B cells without significantly altering normal B cell function or repertoire.

The most widely held model for latency establishment posits that gammaherpesviruses have evolved mechanisms to mimic natural B cell activation pathways, such that infection of naïve follicular B cells results in their activation and subsequent differentiation to memory B cells [4]. The model contends that lifelong infection is maintained because latent virus is indefinitely retained in this long-lived pool of circulating, resting memory B cells. Work from Thorley-Lawson's group has provided important *in vivo* support for this concept by demonstrating that in chronically infected individuals EBV genome is maintained in a frequency of circulating memory B cells that, while variant among individuals, remains stable over time, suggesting that B cell homeostatic mechanisms maintain a lifelong latency setpoint [5]. Similarly, during chronic infection MHV68 is primarily restricted to class-switched memory B cells [6], [7] and is maintained at a stable frequency over time [8].

While work with both EBV and MHV68 support the basic concept that virus-driven mature B cell differentiation contributes to lifelong latency, it remains unclear how memory B cell infection is maintained at a steady setpoint. The two most prevalent hypotheses hold that maintenance of the infected memory B cell pool occurs via reactivation of latent virus and reseeding naïve B cells, with subsequent virus-driven differentiation to memory B cells [9], [10], or via homeostatic proliferation, with virus episome replication and segregation to daughter cells [5]. However, one intriguing alternate possibility is that lifelong latency is facilitated by continual infection of newly generated developing B cells, which subsequently follow normal B cell maturation pathways. In support of this concept, newly formed splenic CD21^−^CD23^−^ B cells have been reported to carry MHV68 genome [11], [12], and we have recently demonstrated that developing B cells harboring MHV68 genome are present in both the bone marrow (pro-B/pre-B and immature B) and the spleen (transitional B) throughout chronic infection [13]. However, the lifespan of these cells is only 24 to 72 hours; thus, these findings suggest that (a) MHV68 recurrently infects developing B cells or (b) MHV68 indefinitely extends the life of developing B cells. Because hematopoiesis results in the daily generation of new immature B cells which in turn maintain the mature B cell population [14]–[18], recurrent or stable infection of these early stage cells could allow gammaherpesviruses to continually access the memory B cell compartment.

During B cell maturation, the stochastic process of V(D)J recombination results in randomly generated B cell receptors (BCRs) on developing B cells. To guard the host against the generation of functional autoreactive mature B cells, immature and transitional B cells must navigate through multiple negative selection checkpoints. In the processes of central tolerance in the bone marrow and peripheral tolerance in the spleen, B cells that react with self-antigen are eliminated through apoptotic clonal deletion, are made anergic, or are subjected to further BCR editing [19]–[25]. BCR binding to self-antigen triggers the apoptotic death of immature and transitional B cells due in part to the low expression of host anti-apoptotic proteins Bcl-2, Bcl-XL and A1 in these cells [26]–[31]. Consistent with this, enforced expression of host Bcl-2 or Bcl-XL *in vivo* allows the survival of autoreactive immature and transitional B cells [25], [32]–[34].

Notably, several gammaherpesviruses encode orthologs of host anti-apoptotic proteins. For example, EBV BHRF1, EBV BALF1, and KSHV ORF16 all encode proteins with homology to Bcl-2, and KSHV K13 encodes a protein with homology to the human FLICE inhibitory protein (FLIP) [35]–[39]. Similarly, MHV68 M11 encodes a Bcl-2 ortholog (vBcl-2) [40], [41] that is expressed during latency [12], [42]. While the specific molecular role that MHV68 vBcl-2 plays in B cell infection has not been determined, it is capable of blocking Fas-, TNFα-, Sindbis virus- and dexamethasone-induced apoptosis [41], [43]–[45], as well as rapamycin- and starvation-mediated autophagy [46], [47]. *In vivo*, MHV68 M11 mutants have demonstrated no significant defects during acute infection and only minor defects during latency [43], [48]–[50]. Thus, EBV, KSHV and MHV68 all encode vBcl-2 orthologs that retain anti-apoptotic activity. However, it is unknown whether any of these proteins play a role in promoting the survival of developing B cells.

To further define the role that gammaherpesvirus Bcl-2 orthologs play during chronic infection, we tested whether MHV68 vBcl-2 can promote the survival of developing B cells that undergo BCR-mediated selection. Using MHV68 mutant viruses, we found that vBcl-2 played a critical role in infection of immature and transitional B cells *in vivo* which could be complemented by host Bcl-2. Further, we found that ectopically-expressed vBcl-2 protected immature B cells from BCR-mediated apoptosis. Finally, by depleting developing B cells during chronic MHV68 infection *in vivo*, we uncovered a role for developing B cells in the lifelong maintenance of MHV68 latency in circulating mature B cells.

## Results

### vBcl-2 is required for infection of developing B cells that undergo BCR-mediated clonal deletion

**Figure 1:**
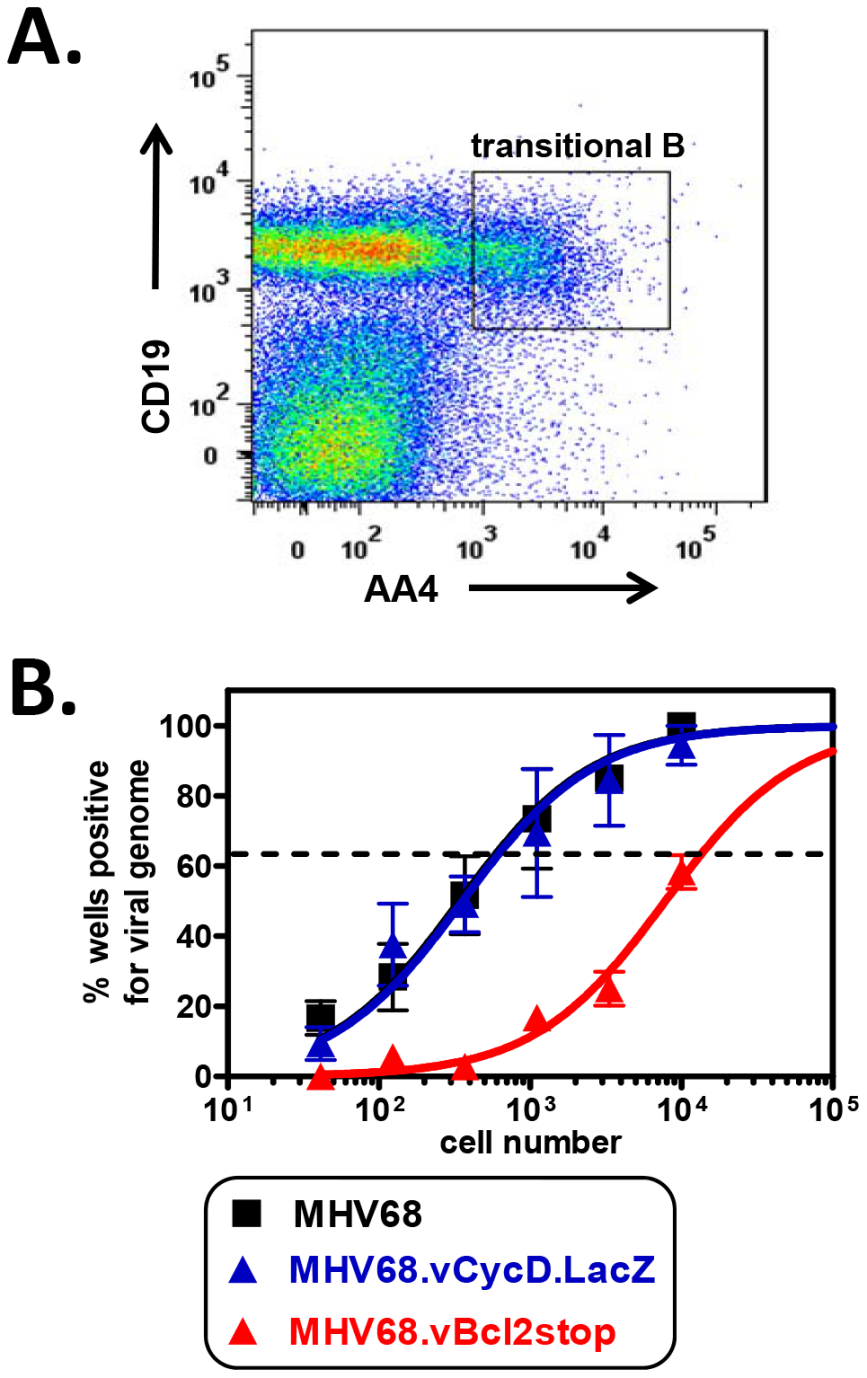
vBcl-2 is required for transitional B cell latency. B6 mice were infected i.n. with 10^4^ PFU wild-type MHV68, MHV68.vBcl-2stop or MHV68.vCycD.LacZ, and spleens were harvested at 16 days post-inoculation. (**A**) Flow cytometric cell sorting was performed to isolate purified transitional B (CD19^+^AA4^+^) cells (post-sort purity analyses of transitional B cell (CD19^+^AA4^+^) populations were performed for each sort and are listed in Table S3). (**B**) Limiting dilution nested PCR for viral genome was utilized to determine the frequency of cells that harbored MHV68 DNA. The frequency of cells positive for viral genome was calculated by Poisson distribution analysis of mean data (n = 3 experiments, 5 mice pooled per group per experiment), as indicated by the dashed line at 63.2%, which is the point at which one viral genome-positive cell per reaction is predicted to occur. *X*-axis is the numbers of cells per reaction, *Y*-axis is the percentage of 12 reactions positive for viral genome.

Clonal deletion of self-reactive developing B cells is one of the core mechanisms utilized to enforce B cell tolerance. Transitional B cells are thought to be the primary target of clonal deletion *in vivo*, with this process resulting in the apoptotic death of a significant portion of the transitional B cell population [15], [24], [27], [30], [51]. Previous work from our laboratory has demonstrated that, following inoculation of wild-type mice, MHV68 maintains latency in a stable frequency of transitional B cells throughout chronic infection [13]. Because these cells have a high rate of turnover and undergo BCR-mediated negative selection, we questioned whether the virus could provide surrogate anti-apoptotic signals to facilitate the survival of infected cells. To test whether the MHV68 vBcl-2 protein played a role in transitional B cell infection, C57BL/6J (B6) mice were inoculated with either wild-type MHV68 or a MHV68 mutant virus deficient in vBcl-2 expression (MHV68.vBcl2stop), and the frequency of latently infection transitional B cells was assessed by PCR. At day 15 post-intranasal (i.n.) inoculation, spleens were harvested and total CD19^+^AA4^+^ transitional B cells were isolated using flow cytometry (Fig. 1A). Although the transitional B cell population in wild-type mice can be further subclassified into T1, T2 and T3 B cells based on surface CD21 and CD23 expression [15], we have previously demonstrated that all three populations are infected by MHV68 [13]; thus for experiments here we examined total transitional B cell infection. At 15 days post-inoculation, both the percentage of transitional B cells in the spleen and the absolute number of splenocytes were similar between the two viruses (Table 1). To determine the frequency of cells harboring viral genome, we performed limiting dilution nested PCR analysis, which allows the specific detection of a single copy of viral genome in a background of up to 50,000 uninfected cells [13], [52], [53]. Strikingly, mice inoculated with MHV68.vBcl2stop displayed a 23-fold decrease in the frequency of infected transitional B cells compared to mice inoculated with wild-type MHV68 (MHV68 1 in 590; MHV68.vBcl2stop 1 in 13,500) (Fig. 1B). This phenotype was not confined to early latency, as MHV68 vBcl-2 mutants were also attenuated in transitional B cells during long-term latency (Fig. S1).

**Table 1:** Percentages and approximate number of B cell populations following infection with wild-type and MHV68 mutant viruses.

Cell population	Virus	a % of total cell suspension	b Approximate cell number
Transitional B cells	WT MHV68	3.4±1.8% (n = 6)	3.37×10^6^
	MHV68.vBcl2stop	3.8±1.1% (n = 3)	3.95×10^6^
	MHV68.vCycD.LacZ	3.7±1.3% (n = 3)	4.00×10^6^
	MHV68ΔBH2	4.2±0.9% (n = 3)	3.99×10^6^
	MHV68Δα1	3.8±1.6% (n = 3)	3.84×10^6^
Mature B cells	WT MHV68	43.7±3.2% (n = 6)	4.33×10^7^
	MHV68.vBcl2stop	37.8±2.6% (n = 3)	3.93×10^7^
	MHV68ΔBH2	42.2±3.3% (n = 3)	4.01×10^7^
	MHV68Δα1	38.2±1.9% (n = 3)	3.86×10^7^
Pro-B/Pre-B cells	WT MHV68	13.8±2.4% (n = 3)	3.86×10^6^
	MHV68.vBcl2stop	14.4±3.1% (n = 3)	3.6×10^6^
Immature B cells	WT MHV68	4.8±1.4% (n = 3)	1.34×10^6^
	MHV68.vBcl2stop	4.5±2.0% (n = 3)	1.13×10^6^

a Percent of total splenocytes that are transitional (CD19^+^AA4^+^) or mature (CD19^+^AA4^−^), and percent of bone marrow cells that are pro-B/pre-B (CD19^+^AA4^+^IgM^−^) or immature (CD19^+^AA4^+^IgM^+^) B cells.

b Estimated total number of transitional, mature, pre-pre, and immature B cells per mouse based on the numbers of cells harvested and the average percentage of analyzed cells in each population.

Although we postulated that the large reduction in MHV68.vBcl2stop-infected transitional B cells was due to the inability of this mutant virus to block BCR-mediated apoptosis, an alternative hypothesis was that the reduction instead resulted from a low level of virus reactivating from latency – a process that is known to be reduced in MHV68 vBcl-2 mutants [49], [50] and could conceivably be critical for re-seeding the transitional B cell population. To control for this possibility, we performed identical experiments using a MHV68 mutated in the viral cyclin D ortholog (vCycD). Like the vBcl-2 mutant, the vCycD mutant virus (MHV68.vCycD.LacZ) undergoes normal acute replication and latency establishment in whole splenocytes and peritoneal cells, but exhibits a low efficiency of reactivation from latently infected cells [50], [54]. However, in contrast to the vBcl-2 mutant virus, the frequency of transitional B cells infected with MHV68.vCycD.LacZ was similar to that of wild-type MHV68 (MHV68 1 in 590; MHV68.vCycD.LacZ 1 in 620) (Fig. 1B), demonstrating that the decreased frequency of infected transitional B cells in the absence of vBcl-2 is not due to decreased infection secondary to reactivation.

**Figure 2:**
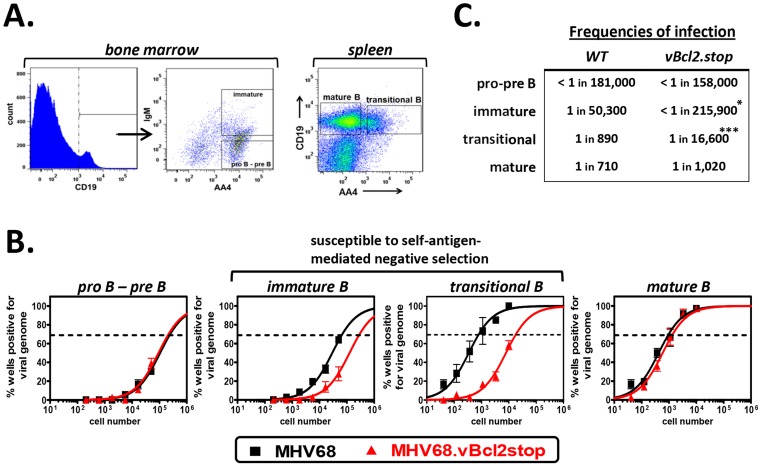
MHV68.vBcl2stop is attenuated in B cells undergoing negative selection. B6 mice were infected i.n. with 10^4^ PFU wild-type MHV68 or MHV68.vBcl-2stop. Sixteen days post-inoculation bone marrow and spleens were harvested from five mice per sample group in each experiment. (**A**) Flow cytometric cell sorting was used to isolate purified pro-B/pre-B (CD19^+^AA4^+^IgM^−^) and immature B (CD19^+^AA4^+^IgM^+^) cells from the bone marrow, and transitional B (CD19^+^AA4^+^) and mature B (CD19^+^AA4^−^) cells from spleens (post-sort purity analyses of transitional B cell (CD19^+^AA4^+^) populations were performed for each sort and are listed in Table S3). (**B**) Limiting dilution nested PCR for viral genome was utilized to determine the frequency of cells that harbored MHV68 DNA. The frequency of cells positive for viral genome was calculated by Poisson distribution analysis of mean data (n = 3 experiments), as indicated by the dashed line at 63.2%, which is the point at which one viral genome-positive cell per reaction is predicted to occur. *X*-axis is the numbers of cells per reaction, *Y*-axis is the percentage of 12 reactions positive for viral genome. Transitional B cell data from MHV68.vBcl2stop sample group is repeated from Fig. 1B for comparison. (**C**) Table comparing frequencies of infection for B cell populations isolated from wild-type MHV68- or MHV68.vBcl2stop-infected mice. *P<0.05, ***P<0.005.

Together these data demonstrated that MHV68 vBcl-2 is critical for latent infection of transitional B cells *in vivo*, and suggested the possibility that vBcl-2 could promote the survival of transitional B cells that are induced to undergo apoptosis as a result of BCR-mediated negative selection. To further explore this possibility, we determined whether loss of MHV68 vBcl-2 expression similarly reduced infection of other B cells that are subjected to BCR-mediated selection events. During early bone marrow hematopoiesis, pro-B and pre-B cells undergo immunoglobulin gene rearrangement and thus do not express a completed cell surface BCR and are not subjected to BCR-mediated selection. In contrast however, in the final stage of bone marrow hematopoiesis immature B cells, which have completed the process of immunoglobulin gene rearrangement, express complete cell surface BCRs and are required to pass a key central tolerance selection checkpoint in which cells that recognize self-antigen are susceptible to clonal deletion [17]. To determine whether reduced MHV68.vBcl2stop infection was indeed a feature of cells undergoing BCR-mediated clonal deletion, we quantified infection in B cell subsets that do (immature B, transitional B) or do not (pro-B/pre-B, mature B), undergo selection. Fifteen days after inoculation of wild-type B6 mice with MHV68 or MHV68.vBcl2stop virus, bone marrow cells and splenocytes were harvested, and purified populations of B cells were isolated using flow cytometric sorting (Fig. 2A). Pro-B/pre-B cells (CD19^+^AA4^+^IgM^−^) and immature B cells (CD19^+^AA4^+^IgM^+^) were isolated from the bone marrow, and transitional B cells (CD19^+^AA4^+^) and mature B cells (CD19^+^AA4^−^) were isolated from the spleen. Total bone marrow and splenocyte cell numbers and percentages of each B cells subset were similar for wild-type and vBcl-2 mutant virus infections (Table 1). The frequency of cells harboring viral genome in each sorted population was determined using limited dilution nested PCR analyses (Figs. 2B and 2C). While loss of vBcl-2 expression had no apparent effect on infection of the pro-B/pre-B cell population, infection of immature B cells and transitional B cells was significantly attenuated in mice inoculated with the vBcl-2 mutant virus (immature 4.3-fold reduced, transitional 23-fold reduced). Furthermore, infection of the bulk mature B cell population in the spleen was not significantly altered in the absence of vBcl-2 expression. Notably, the significantly reduced frequencies of infected immature B cells in the bone marrow and transitional B cells in the spleen was not a reflection of decreased total infection, as the vBcl-2 mutant virus displays near wild-type virus frequencies of infection in bulk splenocytes [50] and bulk bone marrow cells (Fig. S2). Thus, these data demonstrate that vBcl-2 plays a key role specifically in B cell populations that are susceptible to BCR-mediated clonal deletion. Further, these results suggest the possibility that vBcl-2 could promote the survival of MHV68-infected developing B cells and thereby allow those cells to bypass key tolerance selection checkpoints.

### MHV68 infection of transitional B cells requires domains of vBcl-2 that block both apoptosis and autophagy

**Figure 3:**
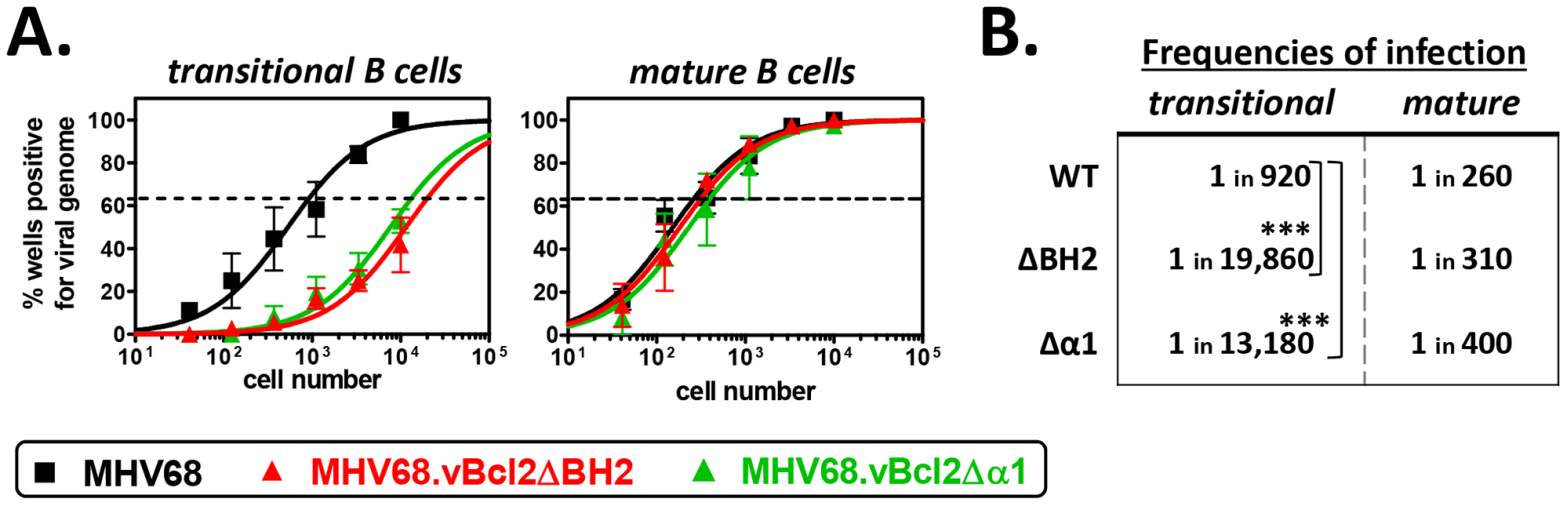
Both BH2 and α1 domains of vBcl-2 are required for efficient infection of transitional B cells. B6 mice were infected i.n. with 10^4^ PFU wild-type MHV68, MHV68.vBcl2ΔBH2 or MHV68.vBcl2Δα1, and spleens were harvested at 16 days post-inoculation. For each sample group in each experiment, splenocytes were pooled from 3–5 mice. Flow cytometric cell sorting was performed to isolate purified transitional (CD19^+^AA4^+^) and mature (CD19^+^AA4^−^) B cell populations, as shown in Figure 2A. (**A**) Limiting dilution nested PCR for viral genome was utilized to determine the frequency of cells that harbored MHV68 DNA. The frequency of cells positive for viral genome was calculated by Poisson distribution analysis of mean data (n = 3 experiments), as indicated by the dashed line at 63.2%, which is the point at which one viral genome-positive cell per reaction is predicted to occur. *X*-axis is the numbers of cells per reaction, *Y*-axis is the percentage of 12 reactions positive for viral genome. (**B**) Table comparing frequencies of transitional and mature B cell infection for each experimental group. ***P<0.005.

Based on the preferential requirement of vBcl-2 in B cells susceptible to clonal deletion, we hypothesized that vBcl-2 blocks BCR-mediated induction of the pro-apoptotic pathway. However, it is notable that in addition to its anti-apoptotic functions, vBcl-2 binds with high affinity to Beclin-1 [46], [47] and blocks the induction of autophagy [46], [47], [49]. Thus, it was conceivable that either or both vBcl-2 functions played crucial roles during infection of developing B cells. Previous work using mutagenesis screens defined independent domains within vBcl-2 that are critical for each function – including a BH2 domain required for anti-apoptotic function and an α1 domain critical for anti-autophagic function – and facilitated the generation of specific loss-of-function MHV68 mutants [49]. To define the requirement of each activity in transitional B cells, we inoculated B6 mice with MHV68, MHV68.vBcl2.ΔBH2 (loss of anti-apoptosis function, normal anti-autophagy function) or MHV68.vBcl2.Δα1 (loss of anti-autophagy function, normal anti-apoptosis function) and performed limiting dilution nested PCR assays on sorted transitional B cells (CD19^+^AA4^+^) and control mature B cells (CD19^+^AA4^−^) at 16 days post-inoculation (Figs. 3A and 3B). In support of our hypothesis, MHV68.vBcl2.ΔBH2 infection of transitional B cells was reduced 21-fold compared to mice infected with wild-type MHV68. Strikingly though, the frequency of transitional B cell infection was similarly reduced (14-fold) in mice infected with MHV68.vBcl2.Δα1. In contrast, the frequencies of mature B cells that harbored viral genome were remarkably similar for all infection groups. Importantly, no significant differences in spleen cell numbers or percentages of B cell populations were observed among groups (Table 1). Thus, these data demonstrate that both the BH2 domain and the α1 domain of vBcl-2 are important for latent infection of transitional B cells, and suggest that actively blocking both apoptosis and autophagy in cells susceptible to clonal deletion is a key facet of MHV68 infection *in vivo*.

### The anti-apoptosis function of vBcl-2 in transitional B cells is complemented in mice that are resistant to negative selection

**Figure 4:**
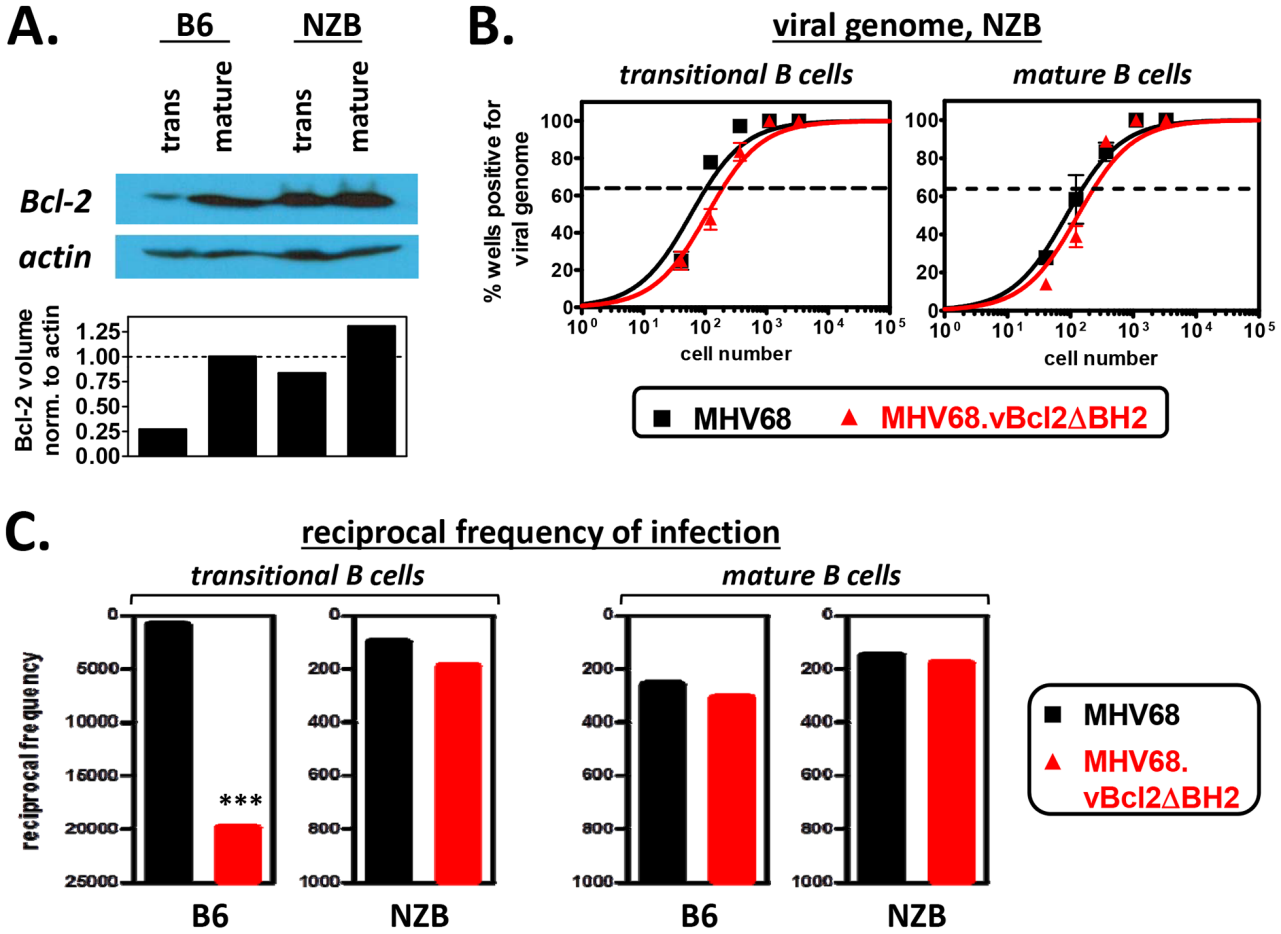
The attenuation of MHV68.vBcl2ΔBH2 in transitional B cells is complemented in mice that are resistant to negative selection. (**A**) Western blots to verify elevated levels of Bcl-2 in NZB transitional B cells. Transitional and mature B cells from B6 or NZB mice were isolated via flow cytometric cell sorting, as shown in Fig. 2A. Whole cell lysates from each sorted cell population were then prepared and subjected to immunoblotting to detect cellular Bcl-2 (26 kDa) and actin (43 kDa). Data shown is representative of 3 individual experiments. (**B**) Limiting dilution nested PCR to determine the frequency cells harboring viral genome. Spleens of NZB mice were harvested at 16 days post -MHV68 or -MHV68.vBcl2ΔBH2 inoculation. For all experiments, splenocytes were pooled from 3 mice per sample group. Flow cytometric cell sorting was performed to isolate purified transitional (CD19^+^AA4^+^) and mature (CD19^+^AA4^−^) B cell populations, as shown in Figure 2A. Limiting dilution nested PCR for viral genome was utilized to determine the frequency of cells that harbored MHV68 DNA. The frequency of cells positive for viral genome was calculated by Poisson distribution analysis of mean data (n = 3 experiments), as indicated by the dashed line at 63.2%, which is the point at which one viral genome-positive cell per reaction is predicted to occur. *X*-axis is the numbers of cells per reaction, *Y*-axis is the percentage of 12 reactions positive for viral genome. (**C**) Comparison of the reciprocal frequencies of infection within the transitional and mature B cell populations of B6 and NZB mice 16 days post-inoculation. Values for the B6 and NZB infection groups are derived from data presented in Figs. 3A and 4B, respectively. ***P<0.005.

The work described above supported the conclusion that a primary function of vBcl-2 during MHV68 infection is to block the apoptosis of developing B cells. This supposition is consistent with data demonstrating that developing B cells are selectively susceptible to apoptosis due to low levels of host Bcl-2 expression [28], [55]. However, one alternative possibility was that MHV68 vBcl-2 mutant viruses are, either directly or indirectly, comprised in their ability to infect developing B cells. To distinguish these two possibilities, we performed complementation experiments in genetically mutated mice that express host Bcl-2 at a higher level than wild-type mice in developing B cells. New Zealand Black (NZB) mice spontaneously develop a lupus-like syndrome, characterized by the production of pathogenic auto-antibodies, in large part because transitional B cells from these mice are resistant to BCR-mediated apoptosis, allowing autoreactive B cells to breach B cell tolerance checkpoints [55], [56]. The resistance of autoreactive transitional B cells to clonal deletion has been shown to correlate with elevated levels of Bcl-2 expression [55]. To confirm this phenotype, we sorted transitional (CD19^+^AA4^+^) and mature (CD19^+^AA4^−^) B cells from the spleens of naïve wild-type B6 and NZB mice and western blotted for host Bcl-2 (Fig. 4A). Indeed, transitional B cells isolated from NZB mice expressed Bcl-2 at a substantially higher level than transitional B cells from B6 mice, and at a level equivalent to B6 and NZB mature B cell populations, which are both resistant to BCR-mediated clonal deletion.

To test whether increased expression of host Bcl-2 in transitional B cells complemented the loss of vBcl-2 anti-apoptotic activity, we performed limiting dilution nested viral genome PCR assays on transitional and mature B cells isolated from the spleens of NZB mice 16 days after inoculation with MHV68 or MHV68.vBcl2.ΔBH2 (Fig. 4B). Interestingly, following inoculation of NZB mice, the frequency of transitional B cells carrying MHV68.vBcl2.ΔBH2 genome was not significantly different from that of wild-type MHV68. Similar levels of infection between the two groups were also observed in mature B cells. These results stand in stark contrast to those from wild-type B6 mice (summarized in Fig. 4C): While the frequency of transitional B cells harboring MHV68.vBcl2.ΔBH2 genome was reduced 21-fold in B6 mice (1 in 920 for MHV68, 1 in 19,860 for MHV68.vBcl2.ΔBH2), the frequency of infection was negligibly reduced in NZB mice (1 in 100 for MHV68, 1 in 190 for MHV68.vBcl2.ΔBH2). It is notable that, consistent with previous reports examining MHV68 infection of lupus-prone mice [57], the frequency of viral genome positive cells was higher for both the transitional and mature B cell populations isolated from NZB mice as compared to B6 mice (Fig. 4C). However, preformed infectious virus was undetectable in splenocytes from NZB mice, demonstrating that this result is not a reflection of enhanced lytic replication in these mice (Fig. S3). Collectively, these results demonstrate that the attenuation of an MHV68 vBcl-2 BH2 mutant virus in transitional B cells can be rescued by host Bcl-2 expression, and accordingly, that the vBcl-2 BH2 mutant is competent for transitional B cell infection. Thus, these results strongly support the hypothesis that the MHV68 vBcl-2 specifically promotes the survival of developing B cell populations that are susceptible to clonal deletion.

### vBcl-2 blocks BCR-induced apoptosis of immature B cells

**Figure 5:**
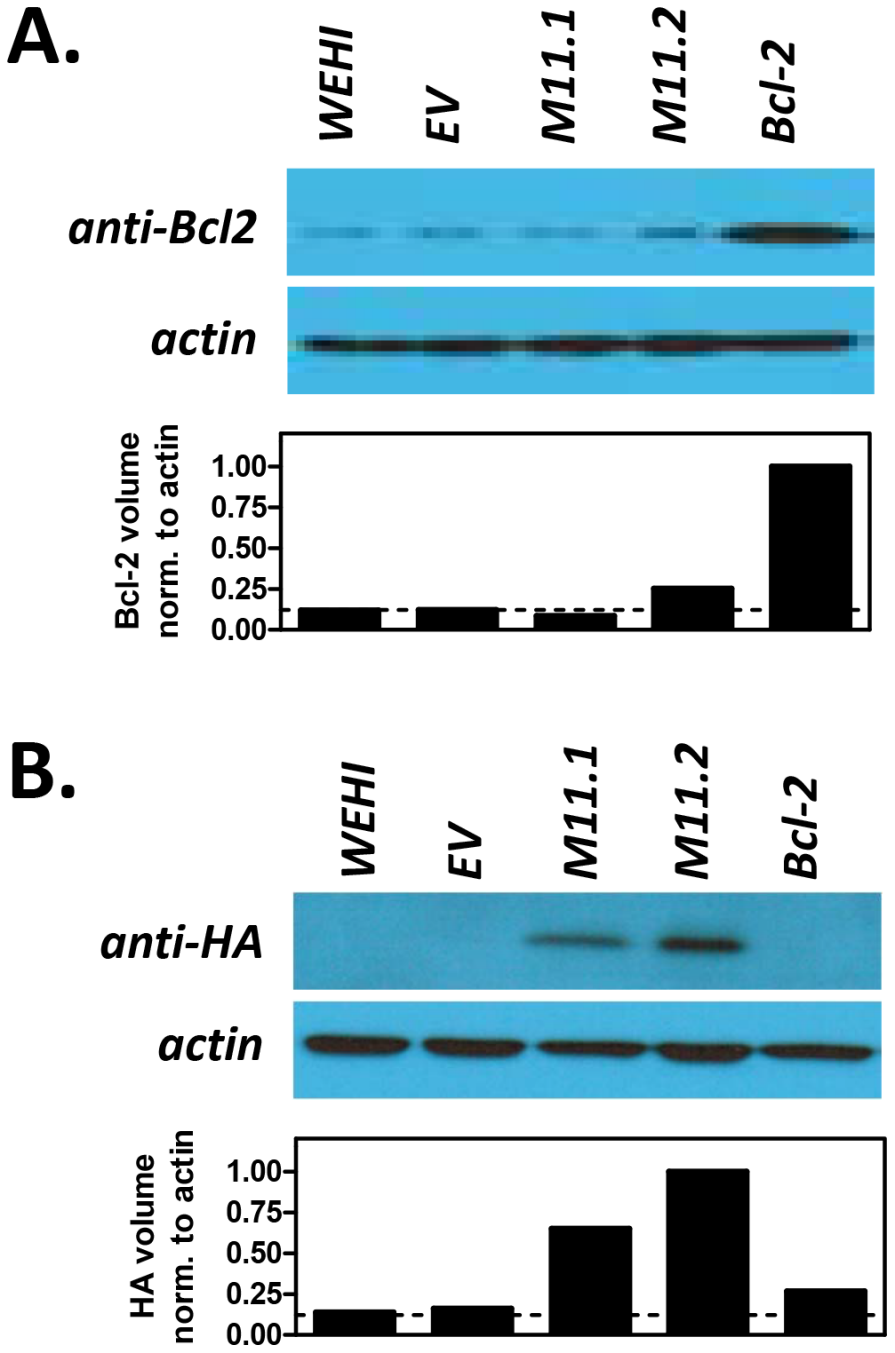
Transduced WEHI-231 B cell lines express host Bcl-2 or viral Bcl-2 (M11) proteins. Parental WEHI-231 cells (WEHI) were transduced with empty retroviral vector (WEHI.EV) or with retroviruses expressing host Bcl-2 (WEHI.Bcl-2) or MHV68 vBcl-2 (WEHI.M11.1 and WEHI.M11.2) to generate stable cell lines. Western blots were performed to confirm expression of (**A**) cellular Bcl-2 or (**B**) viral Bcl-2. Whole cell lysates were collected and subjected to immunoblotting to detect cellular Bcl-2 (26 kDa) and actin (43 kDa) or HA-tagged vBcl-2 (18 kDa) and actin (43 kDa). Data shown is representative of 3 individual experiments.

**Figure 6:**
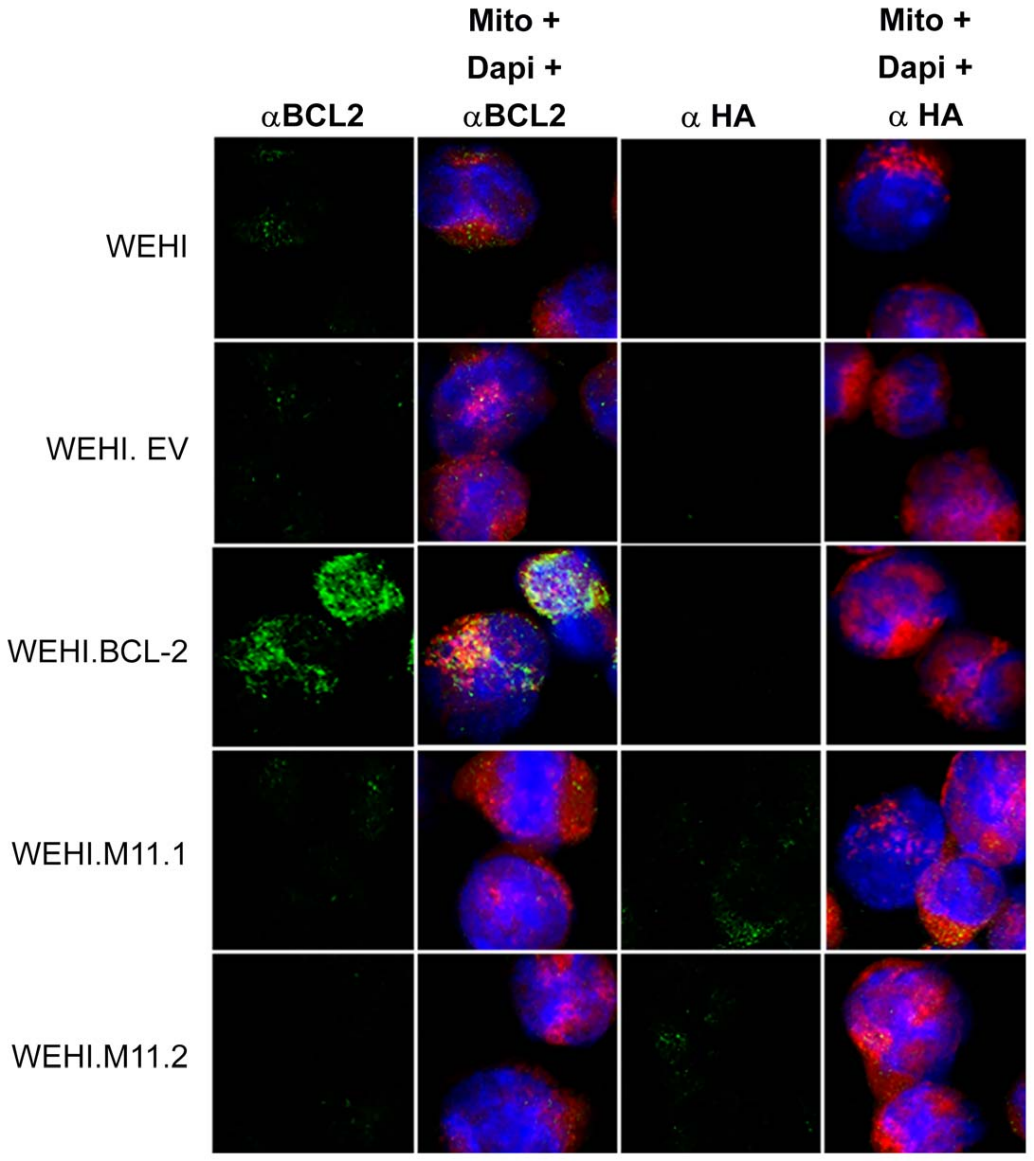
Host Bcl-2 or viral Bcl-2 (M11) expressed by transduced WEHI-231 B cell lines localize to mitochondria. Immunofluorescent staining was performed to visualize expression and cellular localization of cellular Bcl-2 or HA-tagged vBcl-2. WEHI cell lines or control mature A20 B cells were stained with MitoTracker Red to visualize mitochondria (red), primary antibody specific for cellular Bcl-2 or viral Bcl-2 conjugated to HA (green), and DAPI to visualize nuclei (blue). Images were captured with a 63× objective using laser confocal microscopy.

To more directly test whether vBcl-2 could block BCR-mediated apoptosis of immature B cells, we generated stable immature B cells lines that expressed MHV68 vBcl-2 or host Bcl-2. WEHI-231 is a murine IgM^+^ B cell line that displays the phenotype of immature B cells and has been widely used for *in vitro* studies of B cell selection and tolerance mechanisms, including the induction of BCR-mediated apoptosis [58], [59]. To generate WEHI-231 cell lines that ectopically expressed vBcl-2 or host Bcl-2, we engineered murine stem cell viruses (MSCV) that carried genes encoding full-length murine Bcl-2 or full-length MHV68 vBcl-2 fused with a C-terminal HA tag. The MSCV retroviral expression system has been extensively utilized to transduce mammalian cells with target genes of interest [60], [61]. Following generation of recombinant MSCV stocks, viral particles were applied to cultured WEHI-231 cells, and stable cell lines carrying empty vector (EV), host Bcl-2 (Bcl-2), or MHV68 vBcl-2 (M11.1 and M11.2) were generated by antibiotic selection and subcloning. To verify the expression level of transduced genes in the engineered cell lines, we performed western blots on whole cell lysates from each line using antibodies directed toward murine Bcl-2 (Fig. 5A) or HA (Fig. 5B). While the WEHI.Bcl-2 cell line expressed an increased level of Bcl-2 compared to control WEHI-231 cells, Bcl-2 expression in the other cell lines was unchanged, indicating that introduction of other MSCV vectors had no effect on host Bcl-2 expression. Both WEHI.M11.1 and WEHI.M11.2 effectively expressed HA-tagged vBcl-2, although the expression level was slightly increased in the M11.2 line. Similar results were obtained by immunofluorescent microscopy (Fig. 6), demonstrating greatly enhanced expression of host Bcl-2 in the WEHI.Bcl-2 line and ectopic expression of vBcl-2 in both WEHI.M11 lines. Importantly, both Bcl-2 and vBcl-2 localized to mitochondrial compartments, as indicated by co-staining with MitoTracker Red.

**Figure 7:**
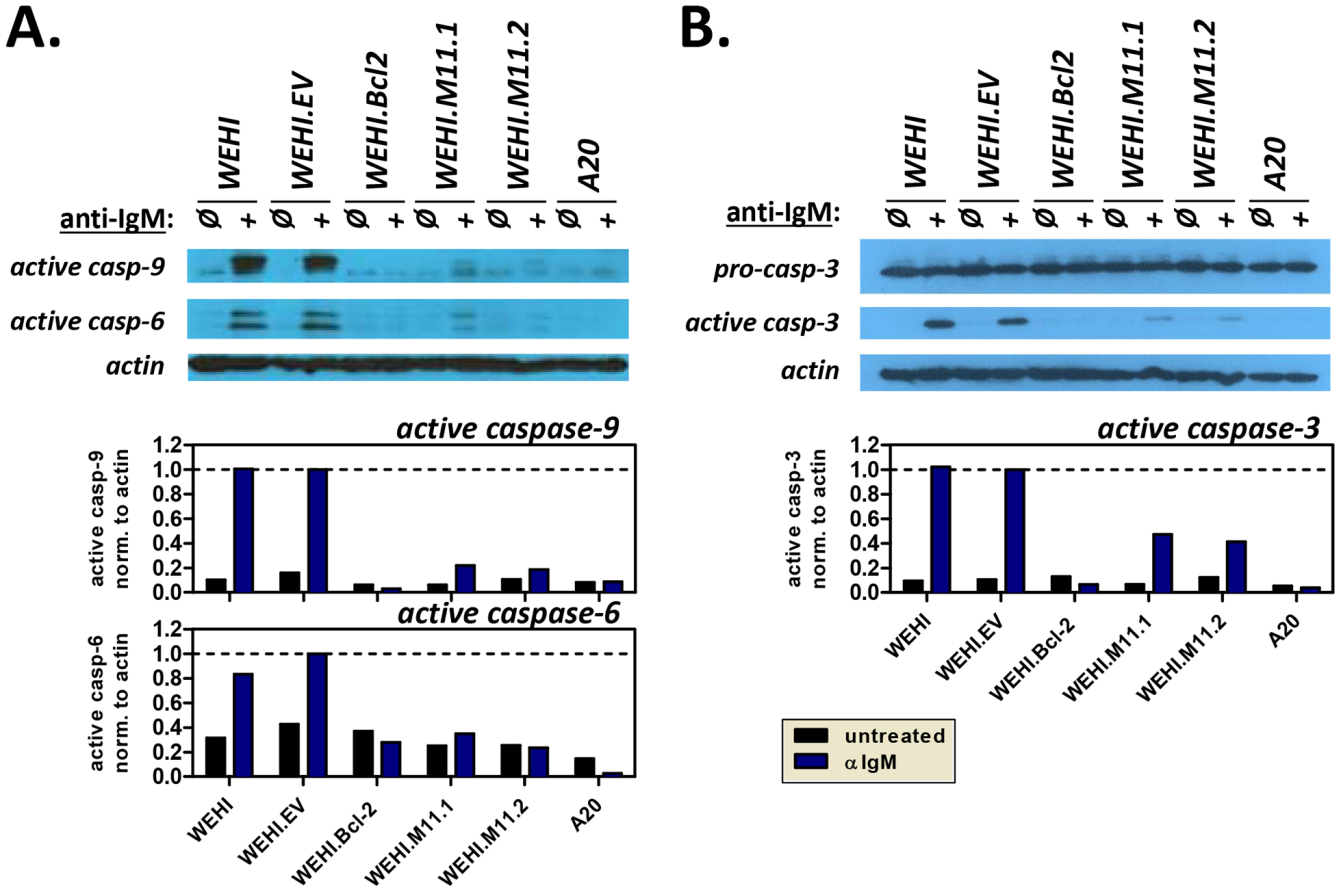
The MHV68 vBcl-2 protects immature B cells against BCR-mediated induction of apoptosome and effector caspase pathway. WEHI immature B cell lines and control A20 mature B cells were plated in duplicate and induced to undergo apoptosis by exposure to anti-IgM (20 µg/ml). Non-induced counterparts for each cell line were included as negative controls. After 16 hours, whole cell lysates were prepared for Western blots or cells were collected for Annexin V staining. Western blots were performed to detect (**A**) the cleaved forms of caspase-9 (37 kDa) and caspase-6 (22 kDa), or (**B**) pro-caspase-3 (35 kDa) and cleaved caspase-3 (17 kDa). Whole cell lysates were collected and subjected to immunoblotting to detect caspases, as well as loading control actin (43 kDa). Densitometry plots indicate the relative volume (arbitrary units) of cleaved caspases after normalization to appropriate actin controls. Data shown is representative of 3 individual experiments.

To test whether vBcl-2 could block the BCR-mediated apoptosis pathway, we cultured each cell line with or without anti-IgM for 16 hours then assayed the cleavage-based activation of the key pro-apoptosis enzymes caspase-9 (Fig. 7A), caspase-6 (Fig. 7A), and caspase-3 (Fig. 7B). Although faint levels of the active, cleaved forms of all three caspases were detectable in unstimulated WEHI cells, their levels were enhanced nearly 10-fold in cells treated with anti-IgM. Because cleaved caspase-9 is a direct downstream product of Apaf-1 oligomerization and apoptosome formation, these results demonstrate that the pro-apoptotic Apaf-1 pathway was induced in BCR-stimulated WEHI cells. Identical results were obtained in the control WEHI line carrying empty vector (EV). In contrast, activation of all three caspases was completely blocked in WEHI cells over-expressing host Bcl-2. Similarly, all three cleaved caspase products were vastly reduced in both WEHI lines expressing vBcl-2 (M11.1, M11.2). As expected, activated caspases were not induced in control A20 mature B cells that express IgG. These results demonstrate that MHV68 vBcl-2 can block BCR-mediated induction of the pro-apoptotic apoptosome/effector caspase pathway in immature B cells.

**Figure 8:**
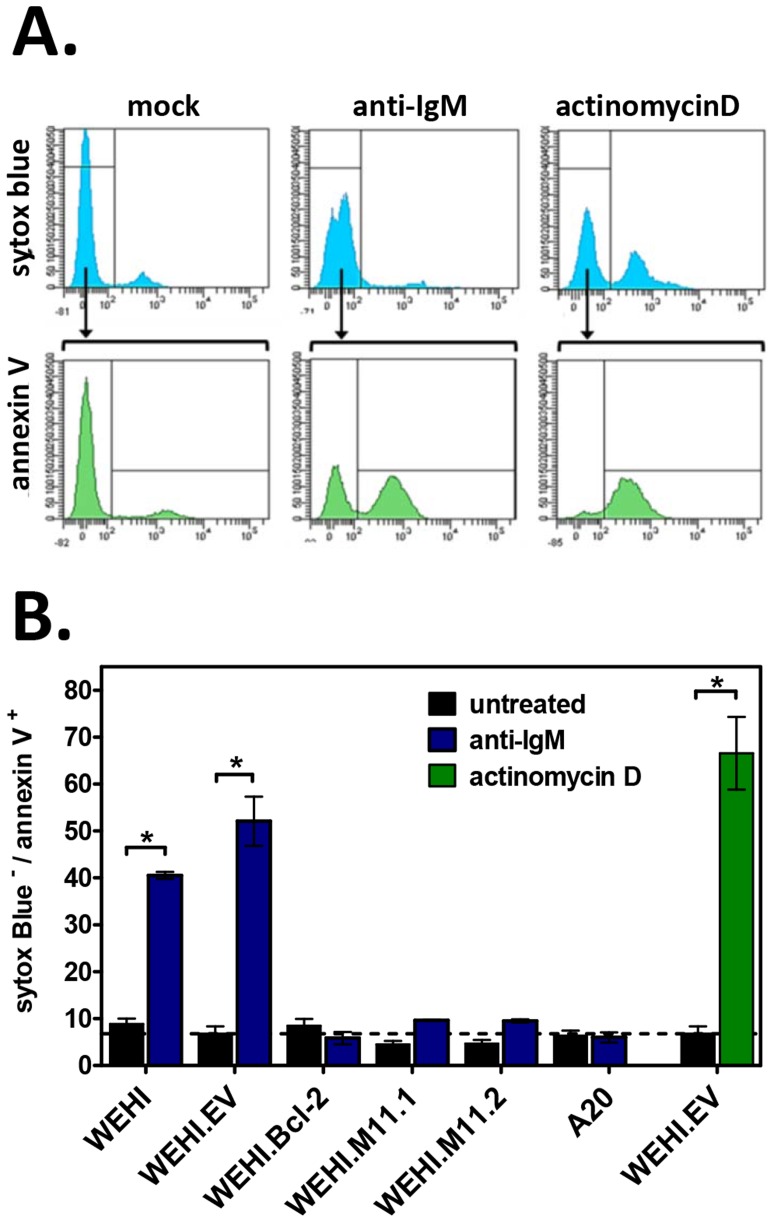
The MHV68 vBcl-2 protects immature B cells against BCR-mediated apoptosis. Flow cytometric analyses were performed to determine the percent of apoptotic cells induced by anti-IgM or positive control actinomycin D (ActD, 40 nM) treatment. Cell samples were double-stained with Annexin V FITC and Sytox Blue, a nucleic acid stain that penetrates cells with compromised plasma membranes. Non-viable cells (Sytox Blue^+^), viable cells (Annexin V^−^ Sytox Blue^−^), and apoptotic cells (Annexin V^+^ Sytox Blue^−^) were detected for all samples as shown. (**A**) Histograms from WEHI.EV samples presented for reference. (**B**) Bar graphs depict the percent of apoptotic cells after treatment with anti-IgM or positive control actinomycin D versus untreated controls. Data presented are the mean ± SD of two experiments with two samples per experiment. *P<0.05.

To further confirm these results, we used annexin V staining to test the ability of vBcl-2 to block the induction of apoptosis at a cellular level. In viable cells, phosphatidlyserine (PS) localizes to the inner face of the plasma membrane, but in the early stages of apoptosis, PS translocates to the outer face of the membrane. Because annexin V binds to PS, the use of fluorescently-labeled annexin V, in conjunction with a dye to detect membrane permeability, provides a convenient means to detect cells undergoing apoptosis. To determine whether vBcl-2 could block the induction of immature B cell apoptosis following BCR signaling, we cultured WEHI cell lines with or without anti-IgM for 16 hours, and then co-stained with annexin V and the nucleic acid stain SYTOX Blue. Actinomycin D (ActD) treatment of WEHI.EV cells was also included as a positive control for induction of apoptosis. ActD inhibits RNA synthesis and is thus a potent inducer of apoptosis via induction of p53 and disruption of mitochondrial membrane potential [62]–[65]. For all cell samples, the percent of cells in early stage apoptosis was quantified by flow cytometric analysis (Fig. 8A). Cells were considered to be in the early stage of apoptosis if they retained an intact membrane (SYTOX Blue^−^) but displayed PS on the cell surface (annexin V^+^). As expected, all nonstimulated WEHI cell lines displayed a low background level of apoptosis (<10%), as indicated by negative staining for annexin V (Fig. 8B). In contrast, following IgM stimulation 40% of WEHI cells and 52% of control WEHI.EV cells were annexin V^+^ and SYTOX Blue^−^, indicating that they were in the early apoptotic stage. These results were similar to the percent of WEHI.EV cells in early apoptosis following ActD treatment (67%), demonstrating that BCR stimulation strongly induced cellular apoptosis in both the parental and empty vector control WEHI cell lines. WEHI cells that over-expressed host Bcl-2 were completely protected from BCR-induced apoptosis (8% for nontreated, 6% for IgM-treated). Similarly, WEHI cells that expressed vBcl-2 were almost completely protected from apoptosis (4% and 5% for untreated M11.1 and M11.2, 9% for IgM-treated). Thus, these data directly demonstrate that MHV68 vBcl-2 can block the induction of BCR-mediated apoptosis in immature B cells.

### Depletion of developing B cells impairs the long-term maintenance of MHV68 latency in mature B cells

Previous work from our laboratory demonstrated that developing B cells carry latent MVH68 throughout chronic infection, implicating this population as a previously unrecognized reservoir for long-term gammaherpesvirus latency [13]. Work presented here further demonstrates that MHV68 vBcl-2 plays a key role in developing B cell infection and that it can block BCR-mediated apoptosis of immature B cells. Because these cells are short-lived and have a high rate of turnover, these findings cumulatively suggest that MHV68 may actively promote the survival of developing B cells in order to take advantage of the normal homeostatic mechanisms that maintain the mature circulating B cell population. In theory, such a strategy would facilitate the recurrent generation of new latently infected mature B cells and thus serve to maintain lifelong latency in the mature B cell compartment. To determine whether recurrent infection of developing B cells is critical for the maintenance of lifelong MHV68 latency, we undertook experiments to deplete developing B cells at the beginning of, or during, a course of MHV68 infection. Because no cell surface markers are known to be solely expressed on developing B cells, we utilized the *in vivo* administration of anti-IL-7 antibody as a means to transiently deplete these cells during MHV68 infection. Interleukin-7 (IL-7) is required for B cell development in the mouse, and in the absence of IL-7 developing B cells do not progress past the pro-B cell stage [66], [67]. In contrast, mature B cells do not require IL-7 for their survival. Thus, transient *in vivo* antibody neutralization of IL-7 results in a marked reduction of developing B cells, including transitional B cells in the spleen, with little or no effect on mature lymphocyte populations [66], [68].

**Figure 9:**
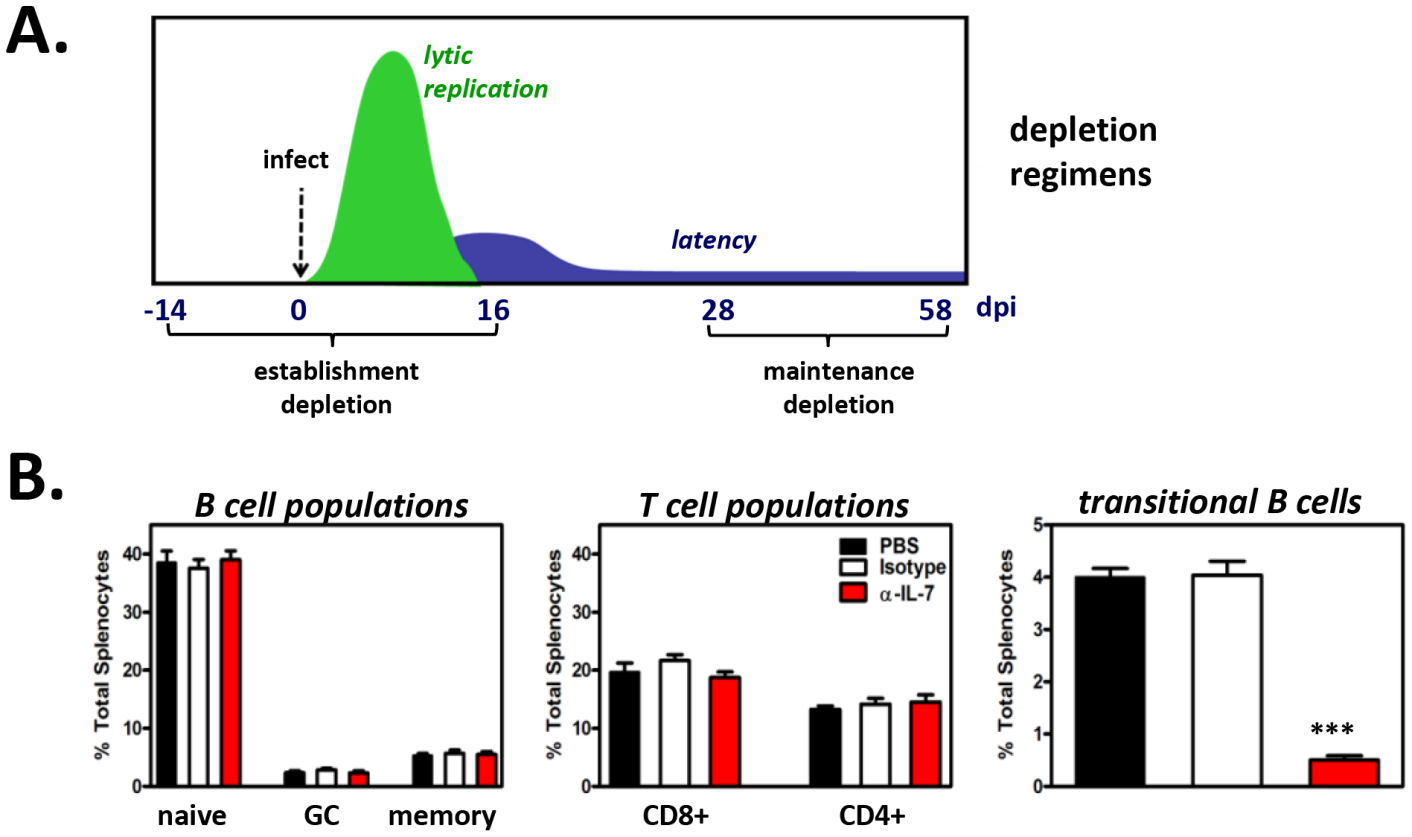
Short-term *in vivo* anti-IL-7 treatment results in depletion of developing B cells but does not alter mature B cell or mature T cell subsets. (**A**) Timeline of antibody treatments and MHV68 inoculation of B6 mice. Starting fourteen days prior to MHV68 inoculation (for depletions during establishment of latency) or 28 days post-MHV68 inoculation (for depletions during maintenance of latency), PBS, isotype control antibody or anti-IL-7 were injected every other day for 4 weeks. Spleens were harvested at 16 dpi or 58 dpi for establishment or maintenance depletions, respectively. Splenocytes from three mice per treatment group were pooled for each experiment. (**B**) Confirmation of developing B cell depletions and preservation of mature lymphocyte populations. A fraction of splenocytes from each treatment group were used for flow cytometric analysis. Bar graph shows the average percent of total splenocytes for B and T cell populations at 58 dpi ± SD (n = 5, splenocytes pooled from three mice per treatment group per experiment). Percentages and approximate number of cells per spleen for each population at 16 and 58 dpi are provided in Tables S1 and S2.

We first examined the contribution of developing B cells to the establishment phase of latency in the mature B cell compartment. Previous reports have shown that 14 day intraperitoneal (i.p.) administration of the murine IgG2b M25 clone of anti-IL-7 neutralizing antibody is sufficient to significantly deplete developing B cells *in vivo* [66]. Thus, for initial experiments, naïve B6 mice were injected i.p. with 2 mg of anti-IL-7 every other day for 14 days. Control mice were injected with 2 mg isotype control antibody (FLAG-M1 IgG_2_b) or PBS. To confirm that developing B cells were effectively depleted but that adaptive immune cells remained, splenocytes from control and anti-IL-7-treated mice were analyzed at the end of the depletion period. While the percentage of mature B (CD19^+^AA4^−^) and mature T (CD4^+^ or CD8^+^) cells were remarkably similar across treatment groups, we observed an 84% depletion (3.8% isotype, 0.6% anti-IL-7) of the transitional B cell population following two weeks of anti-IL-7 treatment (Fig. S4). At this time point, the remaining mice in each group were inoculated i.n. with 10^4^ PFU MHV68. To prevent renewed generation of developing B cells following infection, every other day anti-IL-7 treatments were continued for the final 15 days of the experiment. By 15 days post-inoculation, lytic replication is no longer detectable and latency establishment is at its peak (Fig. 9A).

**Figure 10:**
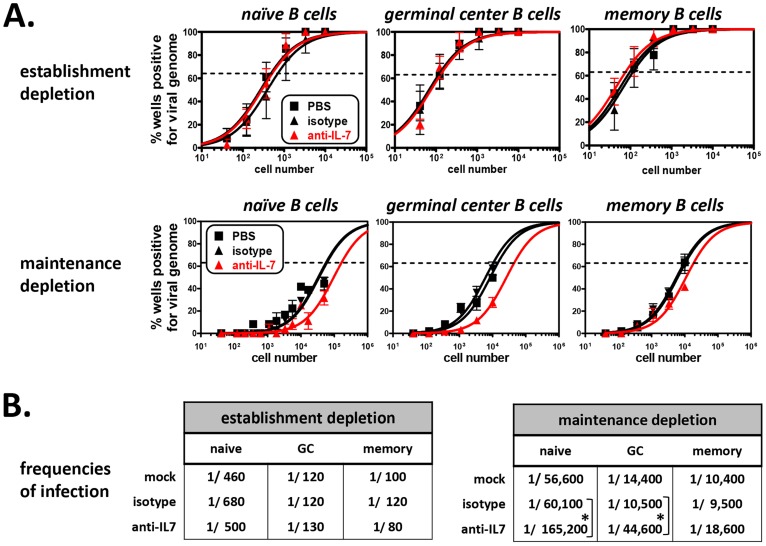
The frequency of MHV68 infection in mature B cells is decreased in mice depleted of developing B cells. (**A**) Limiting dilution nested PCR to determine the frequency of mature B cell subsets that harbor viral genome following antibody depletions. At time of harvest, flow cytometric cell sorting was used to isolate naïve B (CD19^+^AA4^−^IgM^+^), germinal center B (CD19^+^AA4^−^IgM^−^CD38^lo^), and memory B (CD19^+^AA4^−^IgM^−^CD38^hi^) cells (Fig. S4). Limiting dilution nested PCR for viral genome was performed on sorted B cell subpopulations. Top three panels: Depletion during the establishment phase of latency (n = 3). Bottom three panels: Depletion during the maintenance phase of latency (n = 5). The frequency of cells positive for viral genome was calculated by Poisson distribution analysis of mean data, as indicated by the dashed line at 63.2%, which is the point at which one viral genome-positive cell per reaction is predicted to occur. *X*-axis is the numbers of cells per reaction, *Y*-axis is the percentage of 12 reactions positive for viral genome. (**B**) Tables comparing frequencies of infection for mature B cell populations isolated from PBS-, isotype antibody-, or anti-IL-7-treated mice. Frequencies are derived from experiments presented in Panel C. *P<0.05, ***P<0.005.

At experiment termination, splenocytes were harvested and pooled from 3 mice per treatment group, and flow cytometric sorting was performed to isolate naïve (CD19^+^AA4^−^IgM^+^), germinal center (CD19^+^AA4^−^IgM^−^CD38^lo^), and memory (CD19^+^AA4^−^IgM^−^CD38^hi^) B cells for MHV68 latency analyses. Pooled cell suspensions were simultaneously analyzed to confirm developing B cell depletion. At 15 days post-virus inoculation (a total of 29 days of antibody administration), greater than 91% of splenic transitional B cells were depleted (Table S1), while mature T cell populations (CD4+ T cells, CD8+ T cells) and mature B cell populations (naïve B cells, germinal center B cells, memory B cells) remained normal (Tables S1 and S2). Subsequently, naïve, germinal center, and memory B cell populations were sorted (Fig. S4) and analyzed for the presence of viral genome by limiting dilution nested PCR (Fig. 10A). Despite the nearly complete depletion of developing B cells during early infection, the frequencies of naïve, germinal center and memory B cells harboring viral genome in the anti-IL-7 treatment group were nearly identical to that of mock and isotype control groups (Fig. 10B). Because we did not observe 100% depletion of developing B cells, we cannot preclude with certainty the possibility that a low level of developing B cells is sufficient to impact mature B cell latency. However, the finding that early mature B cell latency was completely unaltered in the absence of greater than 95% of the developing B cell population strongly suggests that developing B cells do not play a significant role in the establishment phase of MHV68 latency. Furthermore, these experiments demonstrate that transient IL-7 depletion does not significantly impact T cell control of MHV68 latency, since it is well-established that loss of T cell effector function results in increased numbers of latently infected cells [52], [69]–[73].

Previous work from our laboratory and others has demonstrated that the establishment phase of latency is fundamentally different from the maintenance phase of latency with regard to infected cell composition and, presumably, the molecular profile of viral gene expression [6]–[8]. Notably though, MHV68 infection is maintained in a stable frequency of immature and transitional B cells over time [13], suggesting that infection of these cells may play a key role in facilitating lifelong latency. To determine whether developing B cells contribute to the maintenance phase of latency in mature B cells, we performed IL-7 depletion experiments after the establishment of chronic infection. For these experiments, B6 mice were infected i.n. with 10^4^ PFU of MHV68, then housed untouched for 28 days, allowing time for the virus to set up stable latency. Beginning on day 28, anti-IL-7 was administered every other day for 30 days (Fig. 9A). At 58 days post-inoculation, splenocytes were harvested and stained, and flow cytometric analysis and sorting was performed. Following this 30 day depletion regimen, the percentage of transitional B cells was reduced greater than 87% (4.0% mock and isotype, 0.5% anti-IL7), but importantly, the percentages and absolute numbers of mature B cells and T cells were unaffected (Fig. 9B and Tables S1, S2). To determine whether developing B cell depletion altered the maintenance of mature B cell latency, we performed limiting dilution nested viral genome PCR on sorted naïve, germinal center and memory B cells from each treatment group (Fig. 10A). As expected, in the control groups the overall frequency of infection in each population dropped significantly from 15 days to 58 days (Fig. 10B), owing to the contraction of the early expansion phase of latency and the establishment of stable long-term infection [7]. Interestingly, short-term depletion of the developing B cell population during the stable maintenance of latency led to a statistically significant decline in the overall frequencies of infection in the naïve and germinal center B cell populations (2.8-fold for naïve, 4.2-fold for germinal center) and a more subtle decline of infection in the memory B cell population (2.0-fold). These data stand in clear contrast to depletions during the establishment phase of latency, suggesting that the maintenance of long-term latency in mature B cells requires a fundamentally different virological process than early stage infection. These results for the first time provide a clear link between infection of developing B cell populations and latency in the mature B cell compartment.

## Discussion

**Figure 11:**
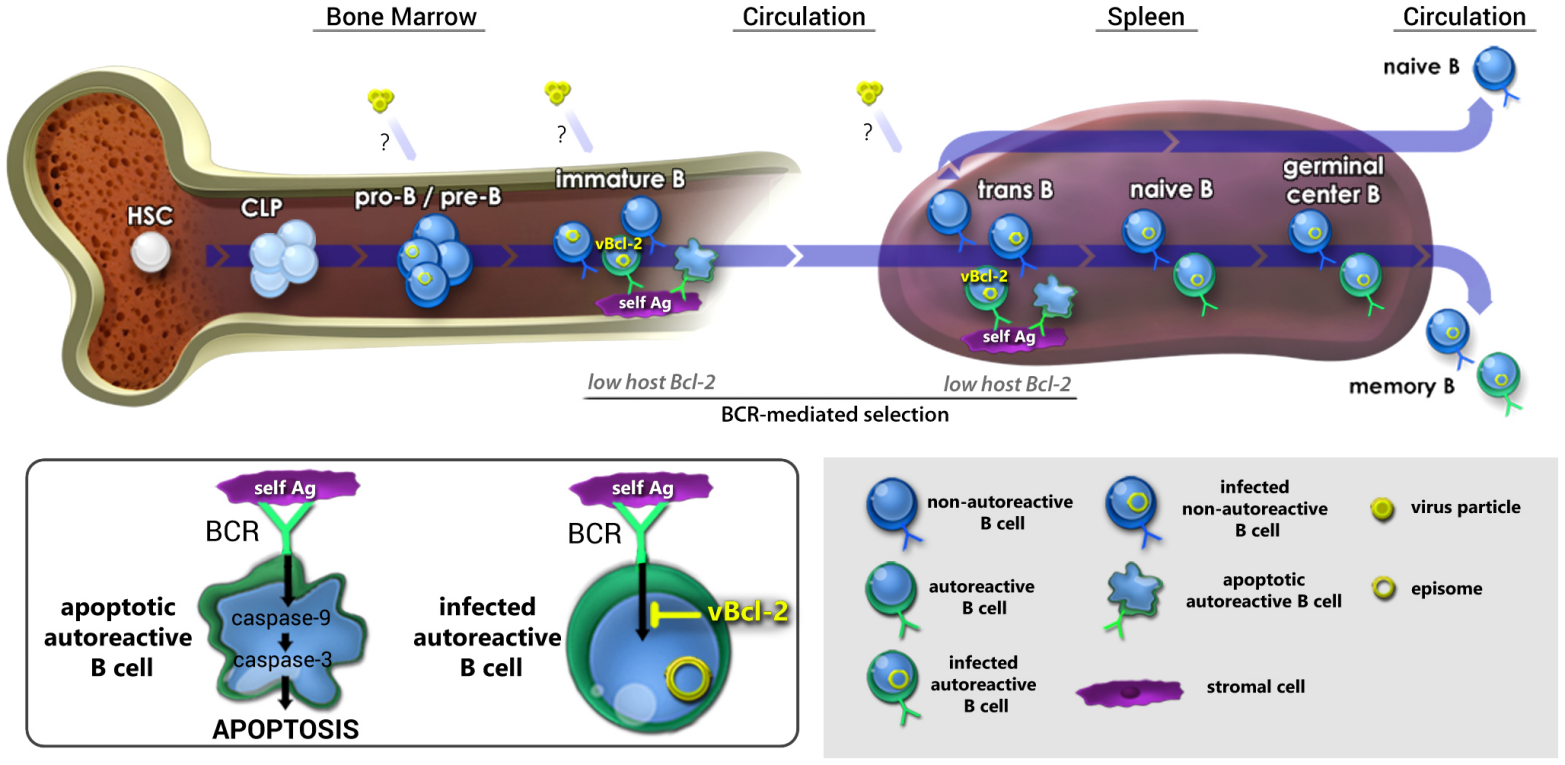
Working model: vBcl-2 promotes the survival of developing B cells and the maintenance of latency in the mature B cell reservoir. Working model for how gammaherpesvirus infection of the self-renewing reservoir of developing B cells may contribute to the long-term maintenance of latency in the circulating mature B cell reservoir. We have previously demonstrated that pro-B/pre-B cells, immature B cells, and transitional B cells carry viral genome throughout chronic infection [13], although it is as yet unclear whether these cells are directly infected at each stage or whether they derive through maturation of an infected early B cell. In work presented here, we demonstrated that vBcl-2 is essential for infection of the majority of immature and transitional B cells, but loss of vBcl-2 could be complemented by increased cellular Bcl-2 expression. Because immature and transitional B cells express low levels of host Bcl-2, cells expressing autoreactive BCRs are subject to clonal deletion via induction of pro-apoptotic effector caspases (inset). Thus, we propose that vBcl-2 provides a key survival signal in infected, autoreactive immature and transitional B cells that compensates for low cellular Bcl-2 expression, thereby allowing these cells to bypass negative selection checkpoints. Conversely, non-autoreactive immature and transitional B cells that carry viral genome would not require vBcl-2 expression to survive and mature. We further demonstrated here that *in vivo* depletion of developing B cells during the maintenance phase, but not establishment phase, of latency leads to a decrease in the frequency of infected mature B cells. Accordingly, we propose that recurrent infection of newly generated developing B cells, vBcl-2-mediated bypass of selection checkpoints, and subsequent natural or virus-driven maturation is critical for long-term maintenance of the primary latency reservoir of circulating memory B cells.

The means by which gammaherpesviruses such as EBV maintain lifelong infection at a stable setpoint in circulating memory B cells is poorly understood. Although the vast majority of human gammaherpesvirus research effort has focused on infection of circulating mature B cells, several clinical studies have reported the presence of EBV and KSHV in bone marrow [74]–[78], suggesting the possibility that gammaherpesviruses can infect B cells that are still in the developmental stage. While these reports have mostly attributed the presence of virus in bone marrow to underlying disease states [77]–[84], one alternate possibility is that gammaherpesviruses utilize infection of bone marrow hematopoietic progenitor cells as one facet of their natural life cycle. A key observation in support of this possibility is our previous finding that stable fractions of short-lived immature and transitional B cells carry MHV68 throughout chronic infection [13]. Based on this finding, we have hypothesized that recurrent infection of developing B cells, and their subsequent differentiation along normal maturation pathways contributes to the maintenance of latency in a stable fraction of memory B cells (Fig. 11).

While such a linear B cell infection and differentiation model is an intriguing possibility, this strategy would seem inefficient unless the virus could provide critical signals to promote the survival of those cells that would otherwise undergo clonal deletion. It is perhaps expedient then that all gammaherpesviruses encode anti-apoptotic proteins, including the Bcl-2 ortholog expressed by MHV68. To test whether the MHV68 vBcl-2 plays a key role in developing B cells, we analyzed *in vivo* infection of developing B cell subsets using MHV68 vBcl-2 mutants and determined whether vBcl-2 alone could block BCR-mediated apoptosis of immature B cells. In work described here, we demonstrate that efficient MHV68 infection of immature and transitional B cells requires vBcl-2 expression. Interestingly though, this gene was not required for infection of the pro-B/pre-B or mature B cell populations, which are not subjected to clonal deletion checkpoints, strongly suggesting that a key role of vBcl-2 *in vivo* is to promote the survival of B cell populations that are susceptible to BCR-mediated selection. Importantly, increased host Bcl-2 expression in transitional B cells complemented the lack of vBcl-2 expression during MHV68 infection, demonstrating that (a) MHV68 vBcl-2 mutants are competent for developing B cell infection, and (b) vBcl-2 carries out functions that are normally associated with host Bcl-2 activity. Consistent with this conclusion, BCR-mediated clonal deletion of developing B cells has been attributed to low Bcl-2 expression [27], [28], [31], [55], [85], and we demonstrate here that expression of vBcl-2 in WEHI-231 immature B cells blocked BCR-induced cleavage of caspases -9, -6 and -3, and induction of cellular apoptosis. Finally, transient *in vivo* depletion of developing B cells during the maintenance phase of long-term latency resulted in reduced numbers of mature B cells carrying viral genome, strongly suggesting that ongoing infection of developing B cells is intimately linked to maintaining latent infection in a stable fraction of circulating mature B cells. Collectively, these studies provide evidence that MHV68 utilizes a Bcl-2 ortholog to promote infection and survival of developing B cells, and point to a key role for developing B cells in the sustenance of lifelong latency (Fig. 11).

### Functional role of vBcl-2 in developing B cells

Host proteins of the Bcl-2 family play a major role in regulating B cell survival, especially during clonal deletion. The pro-apoptotic Bcl-2 family proteins Bak, Bax, and Bim have been shown to mediate the apoptotic death of self-reactive B cells following BCR stimulation with antigen [8], [13], [86]–[88]. Conversely, overexpression of anti-apoptotic proteins Bcl-2 or Bcl-XL prevents BCR-mediated cell death [25], [32]–[34], [55]. Thus, our observations that the MHV68 vBcl-2 mutants displayed a significant defect in immature and transitional B cell infection are consistent with the conclusion that vBcl-2 plays an anti-apoptotic role similar to host Bcl-2 in these cell populations. This conclusion is further supported by our complementation experiments in NZB mice, which showed that increased expression of host Bcl-2 in developing B cells completely negated the attenuated phenotype of the vBcl-2 BH2 mutant virus in these cells. Finally, our experiments using WEHI-231 B cells directly demonstrated for the first time that vBcl-2 could block the apoptosis of immature B cells that were stimulated through the BCR. Thus, we speculate that the decline in frequency of genome positive immature and transitional B cells that occurs in the absence of vBcl-2 expression is due to a decrease in the number of infected cells surviving clonal deletion.

It is noteworthy that our findings do not rule out a role for the anti-autophagy function of vBcl-2 in developing B cells. Indeed, our experiments with the vBcl-2 α1 mutant strongly suggest that MHV68 vBcl-2 blockade of autophagy also plays a critical role in infection of developing B cells. This is consistent with previous reports demonstrating that autophagy of WEHI-231 cells and primary splenic B cells is induced by BCR stimulation [89], and that the autophagy pathway may serve as a backup mechanism for cell death when apoptosis is blocked [90]. A natural extension of this conclusion is that autophagy plays a key and underappreciated role in B cell selection. Thus blocking both apoptosis and autophagy may be requisite for survival of cells that are otherwise destined for clonal deletion.

### Role of developing B cells in the MHV68 life cycle

Our previous demonstration that immature and transitional B cells carry viral genome throughout chronic infection [13] strongly supported a key role for developing B cells during lifelong latency, but did not address whether a linkage exists between developing B cell infection and the peripheral mature B cell latency reservoir. Does infection of developing B cells directly contribute to the dynamic maintenance of latency in circulating mature B cells? Or instead is it an autonomous event, unrelated to peripheral B cell infection? In work presented here, we gained insight into this question by depleting developing B cells *in vivo* then assessing the extent of latent infection in mature B cell subpopulations. Interestingly, developing B cell depletion from 28 to 58 dpi resulted in a pronounced and statistically significant decrease in naïve and germinal center B cell infection, demonstrating that developing B cells are required for the maintenance of peripheral mature B cell latency. Depleted mice also demonstrated a highly reproducible, but smaller decrease in the frequency of infected memory B cells, likely owing to the relative longevity of memory B cells [91]–[94]. Importantly, these results provide the first clear demonstration of a potential direct link between the infection of developing B cells and stability of the major latency reservoir of circulating mature B cells, and strongly suggest that the maintenance of lifelong latency is a dynamic process that involves constant reseeding of the mature B cell reservoir.

Interestingly, depletion of developing B cells prior to and during the first 16 days of infection had no effect on latency in any of the mature B cell subpopulations. These results demonstrate that developing B cells do not contribute to the early establishment of latency in the mature B cell compartment. This finding is notable because it strongly implies that different virological mechanisms operate *in vivo* during establishment versus maintenance of latency. Consistent with this conclusion, the frequency of latent MHV68 infection peaks at 16–20 dpi during the “expansion phase” of latency, then gradually decreases until it reaches a stable level at 42–49 dpi [52], [53], [95], [96]. Similarly, the percentage of latently infected cells that reactivate *ex vivo* is highest at 16 dpi and decreases over time [52], [53]. In light of our results, it is reasonable to speculate that this transition from an active form of latency to a more quiescent form of latency may reflect a conversion from a majority of mature B cells that were directly infected by free virus during acute replication to mature B cells that arose from differentiation of infected developing B cells. Notably, Thorley-Lawson and colleagues have proposed an analogous concept for EBV, wherein direct infection of memory B cells during acute EBV results in an active latency growth program and subsequent cytotoxic T cell targeting, whereas virus-driven maturation of naïve B cells sets up a quiescent latency program that facilitates life-long infection [4]. While further experiments will be required to unravel these complexities, our results clearly indicate that the stable maintenance of long-term latency is a dynamic process that is distinct from early latency and requires an ongoing contribution from developing B cells.

### Relationship to human gammaherpesvirus infections

To date, it is unclear whether human gammaherpesviruses infect developing B cell populations as part of their natural life cycle in healthy individuals. However, both EBV and KSHV genomes have been detected in the bone marrow and progenitor cells of humans in the context of disease. For example, EBV has been detected in the bone marrow of patients with EBV-associated hemophagocytic lymphohistiocytosis (EBV-HLH) [77], [78] and both EBV and KSHV have been detected in the bone marrow of AIDS patients [74], [75]. Further, EBV-associated lymphoproliferative disease following allogeneic bone marrow hematopoietic stem cell (HSC) transplantation is almost always of donor origin [82], [97], [98]. Consistent with the possibility of a progenitor cell source of gammaherpesvirus infection, KSHV has been detected in circulating human CD34^+^ hematopoietic progenitor cells (HPCs) of KS patients [99] and in morphologically immature cells in the bone marrow of transplant recipients [76]. Additionally, several reports have demonstrated the presence of EBV^+^ B cells, presumed to be of progenitor cell origin, arising from long-term human bone marrow cultures of healthy donors [100], [101] and hematologic patients [102]. Cumulatively, these reports provide substantial support for the concept that the human gammaherpesviruses can infect developing B cells in the bone marrow or in circulation. Nevertheless, a great deal of additional work will be required to comprehensively define the role of precursor B cells during a normal course of EBV or KSHV infection.

It is also noteworthy that several recent reports have correlated high numbers of circulating transitional B cells with high EBV loads in patients at risk for the development of EBV-associated B cell lymphomas. For example, it is now widely recognized that EBV and malaria co-infection correlate with a high incidence of endemic Burkitt's B cell lymphoma in African children [103], [104]. Although the synergistic interplay of these two pathogens during oncogenesis is poorly understood, a recent report demonstrated that infants from a malaria-endemic region of Kenya display normal levels of naïve (IgD^+^CD27^−^) and classical memory (IgD^−^CD27^+^) B cells, reduced numbers of non-class switched memory (IgD^+^CD27^+^) B cells, but expanded numbers of immature transitional (CD10^+^CD34^−^) B cells [105]. Interestingly, this population of children also exhibits increased EBV loads in accordance with earlier ages of infection [106]. Likewise, patients with chronic HIV infections frequently display increased EBV loads [107] and are at high risk for the development of EBV-associated B cell lymphoma, and a recent study linked high EBV loads in chronic HIV patients with an increased frequency of circulating immature or transitional B cells [108]. At minimum, these studies provide correlative evidence of a link between high numbers of developing B cells and enhanced EBV infection, and may suggest that transitional B cells serve as a conventional EBV reservoir that greatly expands during particular types of immune dysfunction.

### A link to autoimmune disease?

Our finding that vBcl-2 promotes the survival of immature and transitional B cells during MHV68 infection may provide an important clue to the long-speculated potential link between gammaherpesvirus infections and autoimmune disease. For example, numerous groups have published reports providing circumstantial evidence of a causal relationship between EBV infection and the development of, among others, multiple sclerosis and systemic lupus erythematosus (reviewed in [109], [110]). Nevertheless, this relationship has been seriously questioned due to the incongruous ubiquity of EBV with the relatively rarity of autoimmune diseases. However, on a teleological basis it is reasonable to speculate that if indeed gammaherpesviruses promote survival and maturation of B cells with autoreactive BCRs, then these viruses would also have a means to prevent autoreactive BCRs from signaling as a means to simultaneously protect the host and facilitate long-term latency. Thus, as with gammaherpesvirus-associated tumors, the development of autoimmune disease may represent an anomalous consequence of gammaherpesvirus infection, likely resulting from synergism with disease-promoting secondary factors such as host genetics or pathogen co-infection. In support of the multifactorial nature of any potential link between gammaherpesviruses and autoimmune disease, several conflicting reports have indicated that MHV68 both suppresses [57], [111], [112] and exacerbates [113], [114] murine autoimmune diseases. In light of our demonstration that MHV68 (a) blocks BCR-mediated apoptosis of immature B cells and (b) promotes the survival of developing B cell subpopulations that are known to undergo autoreactive BCR-mediated clonal deletion, a potential link between gammaherpesvirus infection and the survival of B cells with autoreactive BCRs warrants further exploration.

### Summary

The work presented here represents a substantial step forward in the understanding of the *in vivo* role of a gammaherpesvirus-encoded Bcl-2 ortholog. The finding that viruses deficient in vBcl-2 function were most significantly attenuated in those developing B cell populations that are required to surmount tolerance selection checkpoints strongly suggests that the virus alters normal B cell development outcomes as a means to promote long-term survival. Consistent with this conclusion, *in vivo* depletion of developing B cells during long-term latency resulted in reduced infection in mature B cells, supporting the possibility of a direct link between precursor B cell infection and the stability of lifelong latency in circulating mature B cells. A great deal of further work will be required to determine whether a normal course of gammaherpesvirus infection promotes the simultaneous survival and inactivation of autoreactive B cells, and whether in rare scenarios co-factors can play the role of key intermediary between gammaherpesvirus infection of developing B cells and development of autoimmune disease.

## Materials and Methods

### Mice and MHV68 inoculations

Wild-type C57Bl/6J or NZB mice age 7–10 weeks purchased from Jackson Laboratory (Bar Harbor, Main) were used for experiments presented here. Mice were housed at University of Florida in accordance with all federal and university guidelines. Mice were anesthetized with isofluorane and infected intranasally (i.n.) with 10^4^ PFU of virus in 30 µl serum-free DMEM. MHV68 strain WUMS (ATCC VR1465), MHV68.vBcl2stop [50], MHV68.vBcl2ΔBH [49], and MHV68.vBcl2Δα1 [49] were used for inoculations. At indicated time points mice were sacrificed by exposure to inhalation anesthetic.

### Ethics statement

All animal experiments were performed in strict accordance with Federal and University guidelines. Specifically, we adhered to the recommendations in the Guide for the Care and Use of Laboratory Animals of the National Institutes of Health and the American Veterinary Medical Association Guidelines on Euthanasia. The animal protocol was approved by the Institutional Animal Care and Use Committee at the University of Florida (study number 201105767).

### FACS isolation of B cell populations

To obtain splenocyte and bone marrow cell suspensions, spleens, femurs, and tibias were harvested and single cell suspensions were prepared by homogenizing spleens and flushing each bone with 5 mls of DMEM. For spleen samples red blood cell lysis (144 mM NH4Cl and 17 mM Tris, pH 7.2) was carried out for 5 minutes at 37°C prior to staining. Harvested cells were then suspended in blocking buffer (PBS, 5% bovine serum albumin, 10% normal rat serum, and purified anti-mouse CD16/CD32 [Fc block; clone 2.4G2; BD Biosciences]) for 30 minutes on ice prior to staining with antibodies.

#### Bone marrow B cell isolation

Bone marrow cells were stained with rat anti-mouse: CD19-allophycocyanin FITC (clone 1D3; BD Biosciences), AA4-APC (clone AA4.1; eBioscience), and IgM-PE-Cy7 (clone R6-60.2 BD Biosciences). Fluorescent-activated cell sorting was performed to isolate purified pro B – pre B (CD19^+^ AA4^+^ IgM^−^), immature B (CD19^+^ AA4+ IgM^+^), and mature B (CD19^+^ AA4^−^) cell populations.

#### Spleen B cell isolation

Spleen cells were stained with anti-CD19-FITC and anti-AA4-APC and fluorescent-activated cell sorting (FACS) was used to isolate purified transitional (CD19^+^AA4^+^) and mature (CD19^+^AA4^−^) B cells. Purified mature B cell subsets were obtained by staining splenocytes with rat anti-mouse: CD19-FITC, AA4-APC, IgM PE-Cy7, and CD38-PE. Mature B cell subsets were sorted as: naïve B cells (CD19^+^AA4^−^IgM^+^), germinal center B cells (CD19^+^AA4^−^IgM^−^CD38^low^), and memory B cells (CD19^+^AA4^−^IgM^−^CD38^high^). Isotype controls were used for all experiments. Fluorescence minus one controls were also included for any experiments requiring the use of more than two fluorescently labeled antibodies. Post-sort analyses were performed to determine purity of each isolated B cell population (Table S3). Sorts were performed on a FACS Aria II flow cytometer (BD Biosciences).

### Limiting dilution *ex vivo* reactivation assay

Limiting-dilution assays to determine the frequency of transitional and mature B cells reactivating from latency or containing performed infectious virus were performed as previously described [13], [52], [88]. Briefly, transitional and mature B cell populations were serially diluted two-fold and plated onto MEF monolayers in 96-well plates. Twelve dilutions were plated per sample, and 24 wells were plated per dilution. Wells were microscopically scored for cytopathic effect (CPE) following a three week incubation period. To detect preformed infectious virus, parallel samples of mechanically disrupted cells were plated onto MEF monolayers. This process kills >99% of live cells, while leaving preformed infection virus intact. Data points are the average of two or three independent experiments, each consisting of pooled spleens from three mice, and are presented as the percentage of wells per dilution that scored positive for viral CPE+/− standard error.

### Limiting dilution nested PCR (LDPCR) analyses

LDPCR was used to determine the frequencies of cells positive for MHV68 genome, as previously described [13], [52], [115]. Briefly, cell samples were serially diluted threefold in a background of uninfected RAW 264.7 murine macrophages. A total of 1×10^4^ or 5×10^4^ cells were plated in a 96-well PCR plate at 12 wells per dilution. 10, 1, or 0.1 copies of a MHV68 ORF72 plasmid in a background of RAW 264.7 cells, and RAW 264.7 cells only were included on all plates for controls. Cells were lysed with proteinase K at 56°C for 8 hours. Two rounds of nested PCR were then performed using primers specific for MHV68 ORF72, and 195 bp bands resolved using a 3% agarose gel. Unless otherwise indicated, data points are the average of three to five independent experiments, each consisting of pooled spleens from three to five wild-type C57Bl/6 mice. Data are presented as the mean percentage of wells per dilution that were positive for viral genome +/− standard error. On graphs, the dashed line at 63.2% indicates the point at which one viral genome-positive cell per reaction is predicted to occur. The *x* axis shows the numbers of cells per reaction; the *y* axis shows the percentages of 12 reactions positive for viral genome.

### Cell culture

B cell lines WEHI-231, WEHI empty vector, WEHI Bcl-2, WEHI M11.1, WEHI M11.2, and A20 were maintained in RPMI-1640 containing 10% fetal bovine serum (FBS), 100 U/mL penicillin, 100 mg/mL streptomycin, and 0.05 mM 2-mercaptoethanol with 10% CO_2_, at 37°C. 0.025 µg/mL puromycin was added to culture media of WEHI-231 cell lines carrying Murine Stem Cell Virus (MSCV) vectors in order to maintain selection. The MSCV retro viral vector packaging cell line BOSC23 was cultured in complete Dulbecco's modified Eagle's medium (DMEM) supplanted with 10% FCS, 100 U/mL penicillin, and 100 mg/mL streptomycin and incubated at 5% CO_2_, 37°C.

### Production of stably-infected WEHI cell lines

vBcl-2 and host Bcl-2 were cloned into murine stem cell virus (MSCV) retroviral vectors (Clontech). To detect M11 expression, a hemagglutinin (HA) epitope tag was added to the C-terminus of M11. The MSCV Retroviral Expression System (Clontech) was used to generate WEHI empty vector (WEHI.EV), WEHI M11 (WEHI.M11.1 and WEHI.M11.2), and WEHI Bcl-2 (WEHI.Bcl2) cell lines according to the manufacturer's protocols. Briefly, retroviral stocks were generated by transfecting BOSC23 packaging cells with 8 µg DNA (pMSCV M11, pMSCV bcl-2, or pMSCV empty vector) using Lipofectamine2000 (Invitrogen). Viral supernatants were collected at 48 hours and 72 hours post-transfection and filtered through a 0.45 µm nylon filter. The viral supernatants were treated with 6 µg/mL polybrene and were then added to WEHI-231 cells seeded in 60 mm dishes at 1×10^6^ cells per dish. Twenty four hours post-infection 0.025 µg/mL puromycin was added to cultures and selection was carried out continuously. Western Blots and immunofluorescence microscopy, as described below, were used to confirm expression of HA (M11) and Bcl-2.

### Immunofluorescence microscopy

For immunofluorescence assays, 1×10^5^ WEHI cells were washed with PBS and incubated with MitoTacker Red for 30 min at 37°C. Following incubation, cells were washed twice with PBS. Cells were then fixed with ice cold MeOH for 15 min at −20°C, then pelleted and resuspended in 300 µl PBS. The cell suspension was then fixed to microscope slides via cytocentrifugation using a Cytopro cytospin at 400 RPM for 5 min at room temperature. Next, cells were blocked with 5% normal goat serum (NGS) for 1 hour at room temperature to prevent non-specific antibody binding, then incubated with the primary antibodies rabbit anti-BCL-2, rabbit anti-HA or the IgG isotype control in 2% NGS in PBS overnight. Cells were washed three times with PBS and then incubated with secondary antibody Alexa Fluor 488 goat anti-rabbit IgG (Molecular Probes, Inc.) for 1 hour at room temperature, then washed and then treated with DAPI. Images were captured with Leica software using a Leica laser confocal microscope (TCS SP2 ABOS laser scanning spectral confocal) with 63× objective at 4 times zoom.

### Western blots

Cell lysates were prepared using a 1∶1 ratio of PBS and Laemmli buffer with 2-mercaptoethanol and heating for 5 minutes in a boiling water bath. Total protein per µL of sample was quantitated using Thermo Scientific NanoDrop 2000. Equal amounts of total cellular protein from each sample were separated using SDS-PAGE on 15% acrylamide gels, followed by immunoblotting. Membranes were blocked in a 10% milk–TBS–Tween 20 solution, followed by incubation with primary antibody (anti-HA-tag [Cell Signaling Technology], anti-Bcl-2 [Cell Signaling Technology], rabbit anti-caspase-3, -6, or -9 [Cell Signaling Technology], or anti-actin clone c4 [Millipore]). Blots were then incubated with goat anti-mouse IgG HRP (Millipore) or goat anti-rabbit IgG HRP (Abcam). Antibody-labeled protein bands were detected using Western Lightning Plus-ECL Enhanced Chemiluminescence Substrate (Perkin Elmer). Densitometry was performed using Bio-Rad Gel Doc XR system using Quantity One 4.6.9 software (Bio-Rad).

### Annexin V apoptosis analysis

For flow cytometric annexin V assays, WEHI cell lines and A20 cells were plated at 2.5×10^5^ cells/mL with 2 mL total volume in 6 well plates. Cells were incubated for 16 hours in the absence or presence of 20 µg/ml goat anti-mouse IgM, μ chain specific (Jackson ImmunoResearch Laboratories) or 40 nM Actinomycin D (Sigma). Following a 16 hour incubation, cells were washed in cold PBS and resuspended in 100 µl binding buffer, then incubated on ice for 15 minutes with 10 µl of Annexin V-FITC (BD Biosciences). Following Annexin V staining, cells were washed and resuspended in 400 µl binding buffer and 0.5 µl SYTOX Blue (Life Technologies). Cells were incubated an additional 5 minutes at room temperature before analysis on a LSR II (Becton Dickinson) flow cytometer. Data were analyzed using FACS Diva software (Becton Dickinson).

### *In vivo* depletions

#### Antibody purification

Anti-IL-7 (mouse IgG2b anti-human/mouse IL-7, M25) and isotype control (mouse IgG2b, FLAG-M1) monoclonal antibodies from hybridoma culture supernatant fluid were purified on protein A affinity columns, as previously described [68]. Throughout the purification process, care was taken to minimize endotoxin levels; following purification all antibody preparations were tested and found to contain <10 pg of endotoxin per mg of antibody.

#### In vivo depletions

B6 mice were injected i.p. with 2 mg of anti-IL-7, isotype matched control antibody, or PBS every other day, as previously described [68]. For establishment phase depletions, PBS, isotype control antibody, or anti-IL-7 was administered for 14 days. Mice were then inoculated i.n. with 10^4^ PFU MHV68 and treatments were continued for an additional 15 days in order to maintain depletion of the developing B cell populations. Mice were sacrificed one day after the last treatment injection (15 dpi), then spleens were harvested and naïve, germinal center, and memory B cell subsets were isolated by FACS as described above. To insure that transitional B cells were sufficiently depleted and that mature B and T cell populations were not affected by treatments, FACS analyses of B and T cell populations (described below) were performed prior to MHV68 inoculation (14 days of antibody or PBS injections) and at 15 days post-inoculation (29 days of antibody or PBS injections). For maintenance phase depletions, B6 mice were infected i.n. with 10^4^ PFU MHV68. On day 28, PBS, isotype control antibody or anti-IL-7 was injected i.p every other day for 30 days. Mice were then sacrificed one day after the last injection (58 dpi), spleens were harvested, and then naïve, germinal center, and memory B cell subsets isolated by FACS, and FACS analyses of B and T cell populations were performed.

#### FACS analyses of B and T cell populations

Following spleen harvests, single cell suspensions were prepared, red blood cells lysed, and cells were blocked prior to antibody staining. Cells were then stained with anti-CD19-FITC, anti-AA4-APC, anti-IgM-PE-Cy7, and anti-CD38-PE or with anti-CD4-PE and anti-CD8-APC. FACS analysis was used to determine the percentage of total splenocytes for each B cell population [transitional (CD19^+^AA4^+^), naïve (CD19^+^AA4-IgD^+^), germinal center (CD19^+^AA4^−^IgD^−^CD38^lo^), and memory (CD19^+^AA4^−^IgD^−^CD38^hi^) B cells] and T cell populations [CD8 T cells (CD8^+^CD4^−^) and CD4 T cells (CD8^−^CD4^+^) T cells]. Isotype and fluorescence minus one controls were used for the purpose of setting population gates.

### Statistical analyses

FACS data were analyzed using FACSDiva (BD Biosciences) and FloJo. All other data were analyzed using GraphPad Prism software (GraphPad Software, San Diego, CA). The frequencies of cells positive for viral genome, reactivating *ex vivo*, and containing preformed virus were determined from the nonlinear regression analysis of sigmoidal dose response best-fit curve data. Based on Poisson distributions, the frequency at which at least one event in a given population is present occurs at the point where the regression analysis line intersects 63.2%. Calculation of statistical significance was determined by Student's t test of paired cell dilution results.

## Supporting Information

Figure S1 **vBcl-2 is required for long-term transitional B cell latency.** B6 mice were infected i.n. with 10^4^ PFU wild-type MHV68 or MHV68.vBcl2ΔBH2, and spleens were harvested at 42 days post-inoculation. Flow cytometric cell sorting was performed to isolate purified transitional B (CD19^+^AA4^+^) cells. (**A**) Limiting dilution nested PCR for viral genome was utilized to determine the frequency of cells that harbored MHV68 DNA. (**B**) The frequency of cells positive for viral genome was calculated by Poisson distribution analysis of mean data (n = 2 experiments, 5 mice pooled per group per experiment), as indicated by the dashed line at 63.2%, which is the point at which one viral genome-positive cell per reaction is predicted to occur. *X*-axis is the numbers of cells per reaction, *Y*-axis is the percentage of 12 reactions positive for viral genome. *P<0.05.
(TIF)

Figure S2 **MHV68.vBcl2stop establishes latency in the bone marrow at frequencies equivalent to wild-type MHV68.** B6 mice were infected i.n. with 10^4^ PFU wild-type MHV68 or MHV68.vBcl2stop. At 16 dpi, femurs and tibias were harvested and flushed with 10 ml DMEM. For each sample group in each experiment, bone marrow cells from 5 mice were pooled. LDPCR analyses were performed to determine the frequency of infection for whole bone marrow. The frequency of cells positive for viral genome was calculated by Poisson distribution analysis of mean data (n = 3 for all sample groups, 5 mice pooled per sample group per experiment).
(TIF)

Figure S3 **Increased latency in NZB mice is not due to increased lytic replication.** NZB mice were infected i.n. with 10^4^ PFU of wild-type MHV68 or MHV68ΔBH_2_ and spleens harvested at 16 dpi. For each sample group in each experiment, splenocytes from 3 mice were pooled. Limiting dilution *ex vivo* reactivation assays were preformed, as described in materials and methods. The frequency of cells that contain performed infectious virus was calculated by Poisson distribution analysis of mean data (MHV68 n = 2, MHV68.vBcl2ΔBH2 n = 3). *X*-axis shows the numbers of cells per reaction, *Y*-axis shows the percent of 12 reactions positive for cytopathic effect (CPE).
(TIF)

Figure S4 **Percent lymphocyte populations following 14 day anti-IL-7 treatments and prior to MHV68 inoculation.** Naïve B6 mice were intraperitoneally (i.p.) injected with 2 mg of anti-IL-7 every other day for 14 days. Control mice were injected with 2 mg isotype control antibody or PBS. At the end of the treatment period splenocytes from three mice per treatment group were individually analyzed via flow cytometry to confirm anti-IL-7 depletion of transitional B cells and preservation of mature lymphocyte populations. Bar graph shows the average percent of total splenocytes (n = 3 for all treatment groups).
(TIF)

Figure S5 **Example scheme for flow cytometric sorting of mature B cell populations following 30 days of PBS, isotype control antibody, or anti-IL-7 treatment.** Representative flow plots for isolation of naïve B cells (AA4^−^CD19^+^IgM^+^), germinal center B cells (AA4^−^CD19^+^IgM^+^CD38^lo^), and memory B cells (AA4^−^CD19^+^IgM^+^CD38^hi^). Post-sort purities for each B cell population are shown in Table S3.
(TIF)

Table S1 **Percentage and approximate number of B cell populations per spleen for IL-7 depletion experiments.** Percent and approximate number of total splenocytes that were transitional (CD19+AA4+), naïve (CD19+AA4−IgD+), germinal center (CD19+AA4−IgD−CD38lo), and memory (CD19+AA4−IgD−CD38hi) B cells. For 15 dpi, n = 3. For 58 dpi, n = 5.
(TIF)

Table S2 **Percentage and approximate number of T cell populations per spleen for IL-7 depletion experiments.** Percent and approximate number of total splenocytes that were CD4+ and CD8+ T cells. For 15 dpi, n = 3. For 58 dpi, n = 5.
(TIF)

Table S3 **Post-sort purities of sorted B cell populations.** Post-sort percentages averaged for each population at 16 dpi and 58 dpi.
(TIF)
