# Supplementary figures and images for: A Gammaherpesvirus Bcl-2 Ortholog Blocks B Cell Receptor-Mediated Apoptosis and Promotes the Survival of Developing B Cells In Vivo
Source: PLoS Pathog. 2014 Feb 6;10(2):e1003916. doi: 10.1371/journal.ppat.1003916 (PMC3916410; doi:10.1371/journal.ppat.1003916)

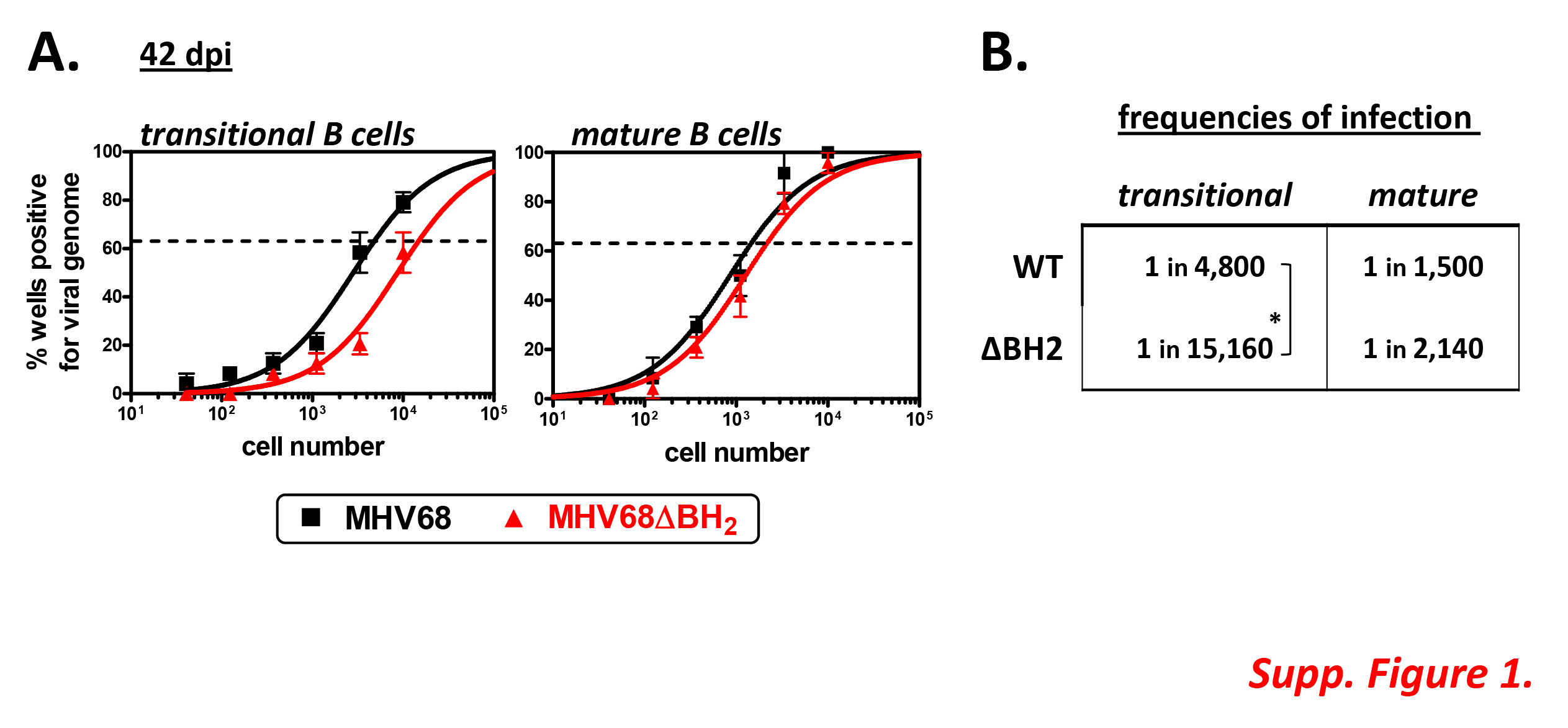

Supplement: Figure S1 — vBcl-2 is required for long-term transitional B cell latency. B6 mice were infected i.n. with 104 PFU wild-type MHV68 or MHV68.vBcl2ΔBH2, and spleens were harvested at 42 days post-inoculation. Flow cytometric cell sorting was performed to isolate purified transitional B (CD19+AA4+) cells. (A) Limiting dilution nested PCR for viral genome was utilized to determine the frequency of cells that harbored MHV68 DNA. (B) The frequency of cells positive for viral genome was calculated by Poisson distribution analysis of mean data (n = 2 experiments, 5 mice pooled per group per experiment), as indicated by the dashed line at 63.2%, which is the point at which one viral genome-positive cell per reaction is predicted to occur. X-axis is the numbers of cells per reaction, Y-axis is the percentage of 12 reactions positive for viral genome. *P<0.05. (TIF) [file ppat.1003916.s001.tif]

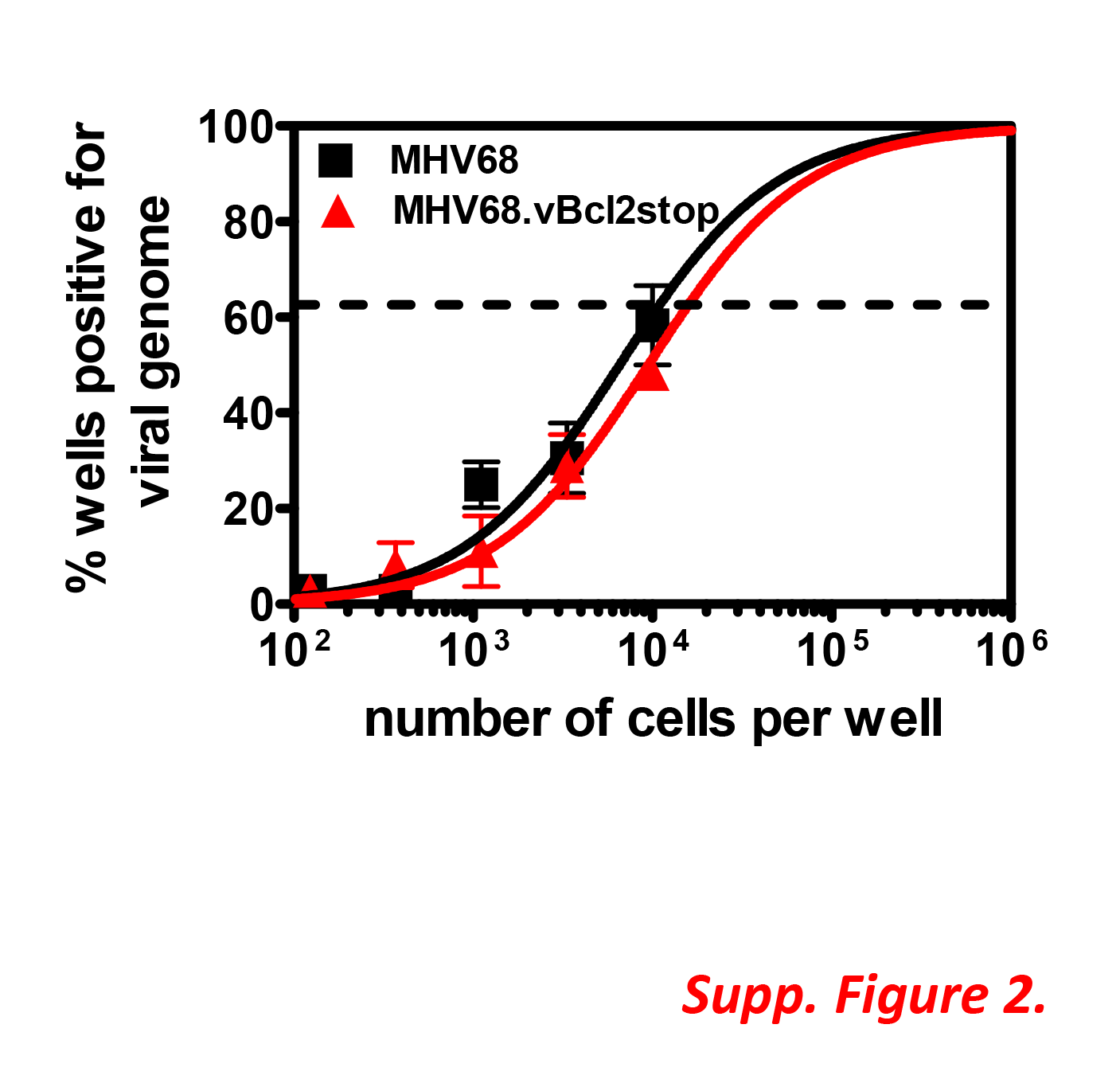

Supplement: Figure S2 — MHV68.vBcl2stop establishes latency in the bone marrow at frequencies equivalent to wild-type MHV68. B6 mice were infected i.n. with 104 PFU wild-type MHV68 or MHV68.vBcl2stop. At 16 dpi, femurs and tibias were harvested and flushed with 10 ml DMEM. For each sample group in each experiment, bone marrow cells from 5 mice were pooled. LDPCR analyses were performed to determine the frequency of infection for whole bone marrow. The frequency of cells positive for viral genome was calculated by Poisson distribution analysis of mean data (n = 3 for all sample groups, 5 mice pooled per sample group per experiment). (TIF) [file ppat.1003916.s002.tif]

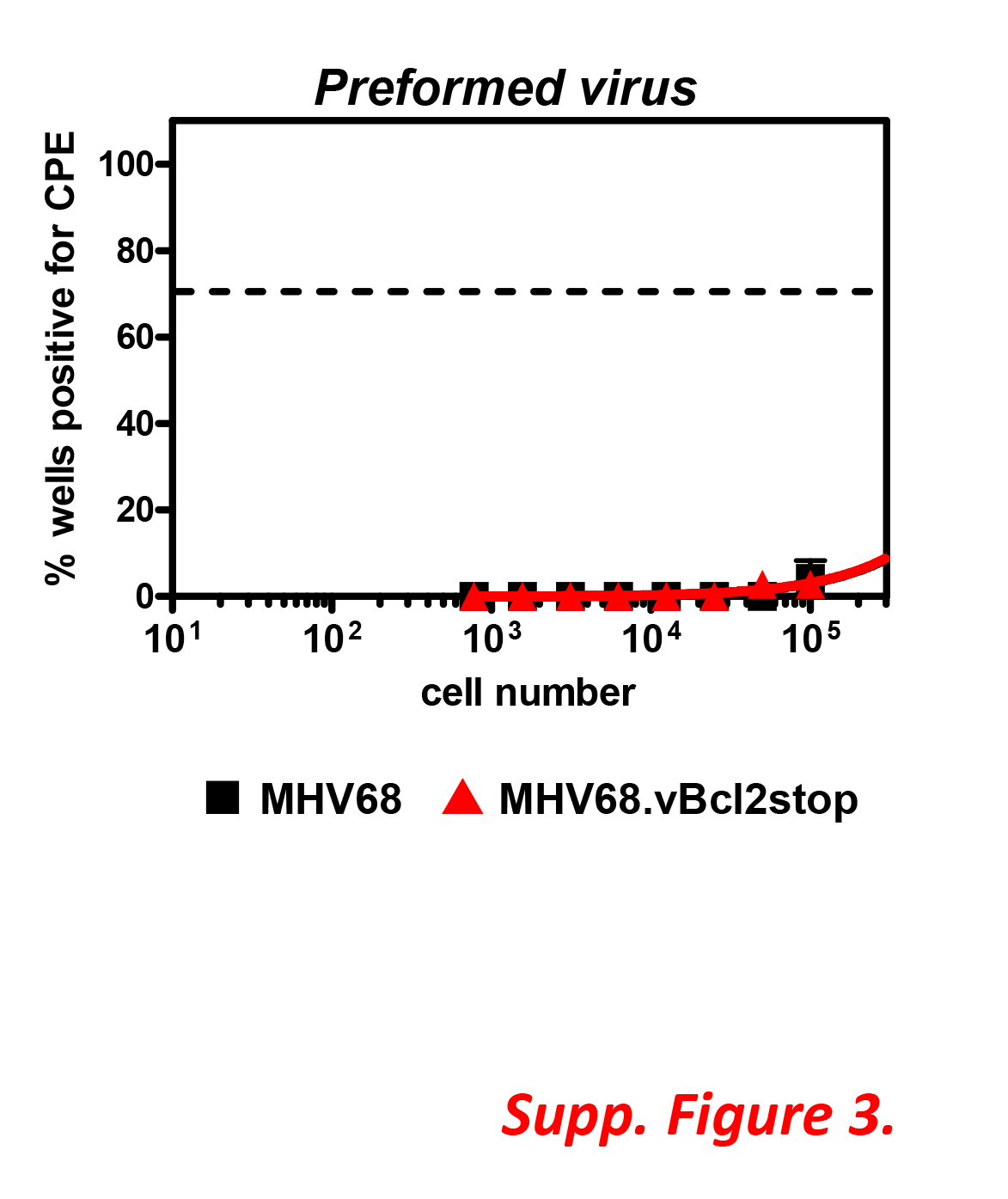

Supplement: Figure S3 — Increased latency in NZB mice is not due to increased lytic replication. NZB mice were infected i.n. with 104 PFU of wild-type MHV68 or MHV68ΔBH2 and spleens harvested at 16 dpi. For each sample group in each experiment, splenocytes from 3 mice were pooled. Limiting dilution ex vivo reactivation assays were preformed, as described in materials and methods. The frequency of cells that contain performed infectious virus was calculated by Poisson distribution analysis of mean data (MHV68 n = 2, MHV68.vBcl2ΔBH2 n = 3). X-axis shows the numbers of cells per reaction, Y-axis shows the percent of 12 reactions positive for cytopathic effect (CPE). (TIF) [file ppat.1003916.s003.tif]

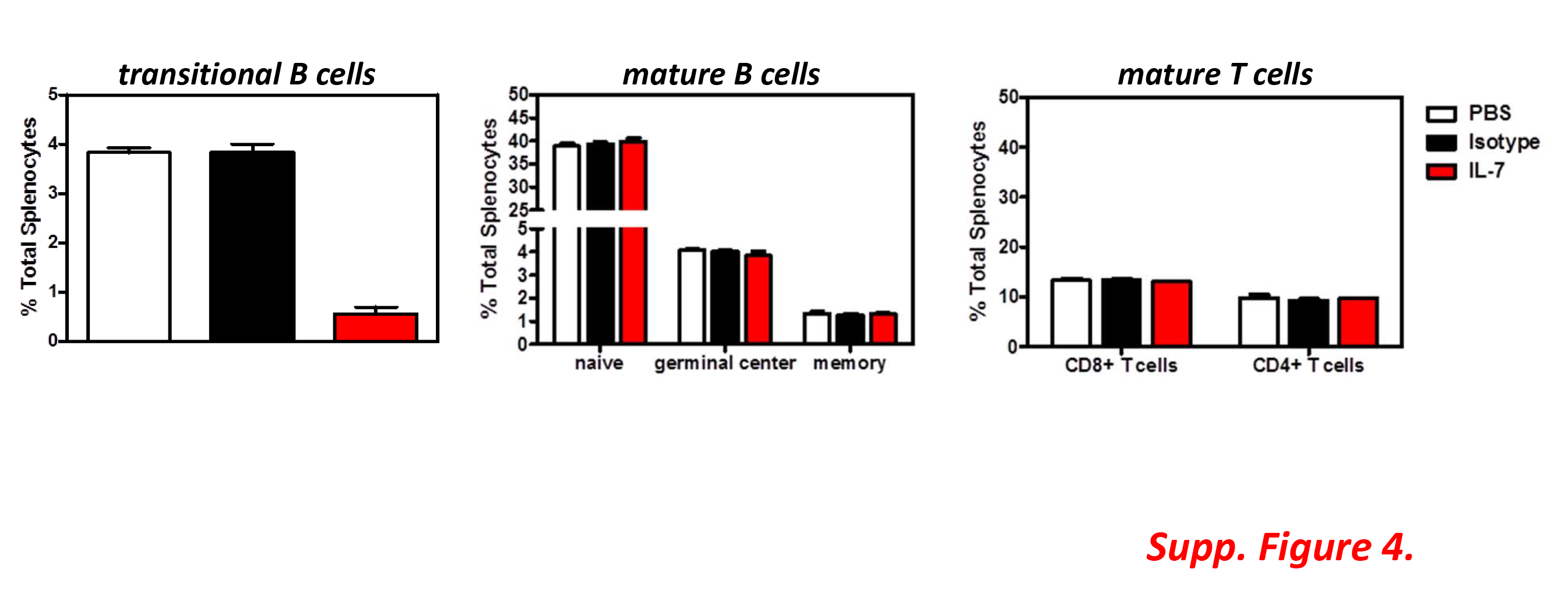

Supplement: Figure S4 — Percent lymphocyte populations following 14 day anti-IL-7 treatments and prior to MHV68 inoculation. Naïve B6 mice were intraperitoneally (i.p.) injected with 2 mg of anti-IL-7 every other day for 14 days. Control mice were injected with 2 mg isotype control antibody or PBS. At the end of the treatment period splenocytes from three mice per treatment group were individually analyzed via flow cytometry to confirm anti-IL-7 depletion of transitional B cells and preservation of mature lymphocyte populations. Bar graph shows the average percent of total splenocytes (n = 3 for all treatment groups). (TIF) [file ppat.1003916.s004.tif]

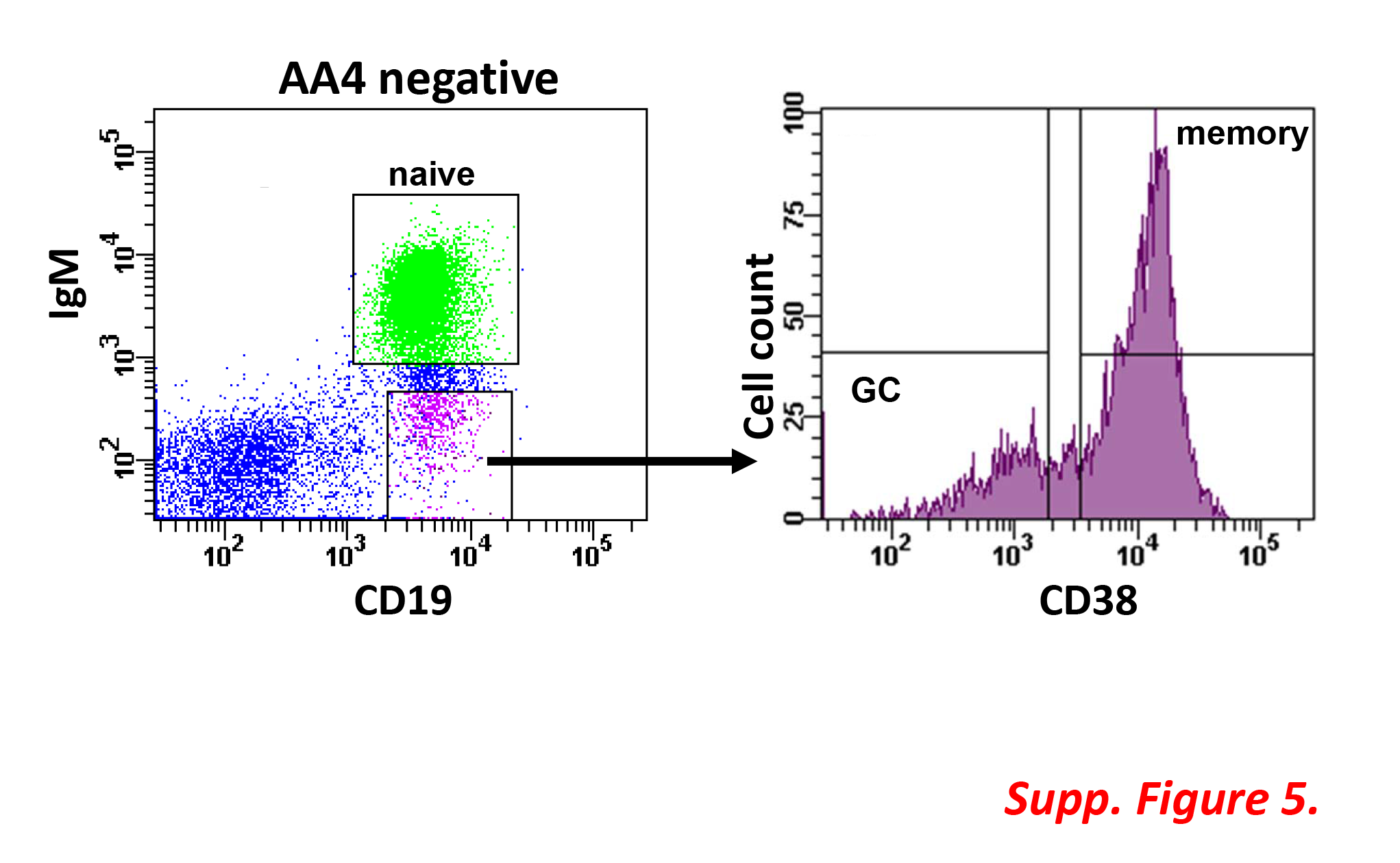

Supplement: Figure S5 — Example scheme for flow cytometric sorting of mature B cell populations following 30 days of PBS, isotype control antibody, or anti-IL-7 treatment. Representative flow plots for isolation of naïve B cells (AA4−CD19+IgM+), germinal center B cells (AA4−CD19+IgM+CD38lo), and memory B cells (AA4−CD19+IgM+CD38hi). Post-sort purities for each B cell population are shown in Table S3. (TIF) [file ppat.1003916.s005.tif]

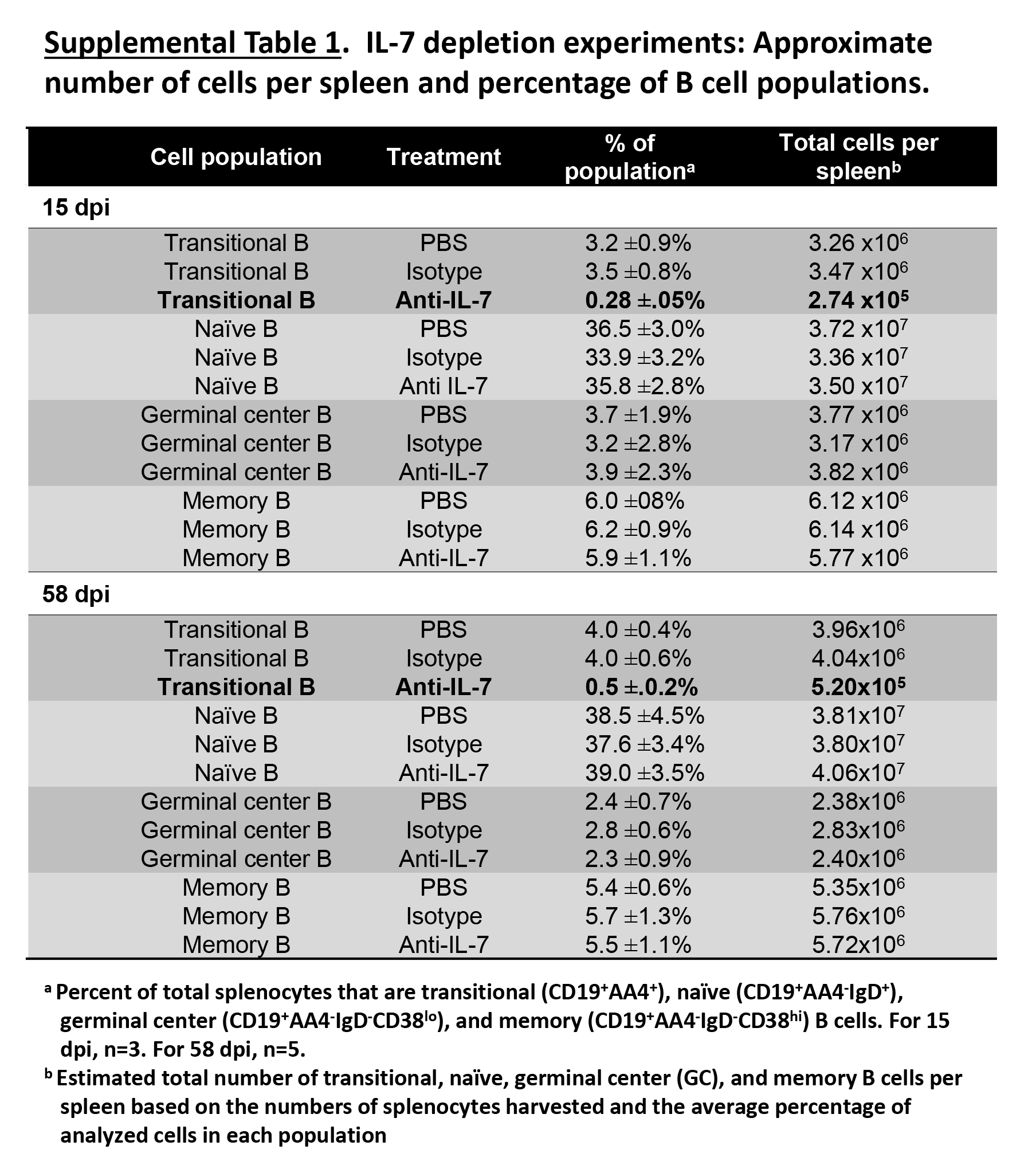

Supplement: Table S1 — Percentage and approximate number of B cell populations per spleen for IL-7 depletion experiments. Percent and approximate number of total splenocytes that were transitional (CD19+AA4+), naïve (CD19+AA4−IgD+), germinal center (CD19+AA4−IgD−CD38lo), and memory (CD19+AA4−IgD−CD38hi) B cells. For 15 dpi, n = 3. For 58 dpi, n = 5. (TIF) [file ppat.1003916.s006.tif]

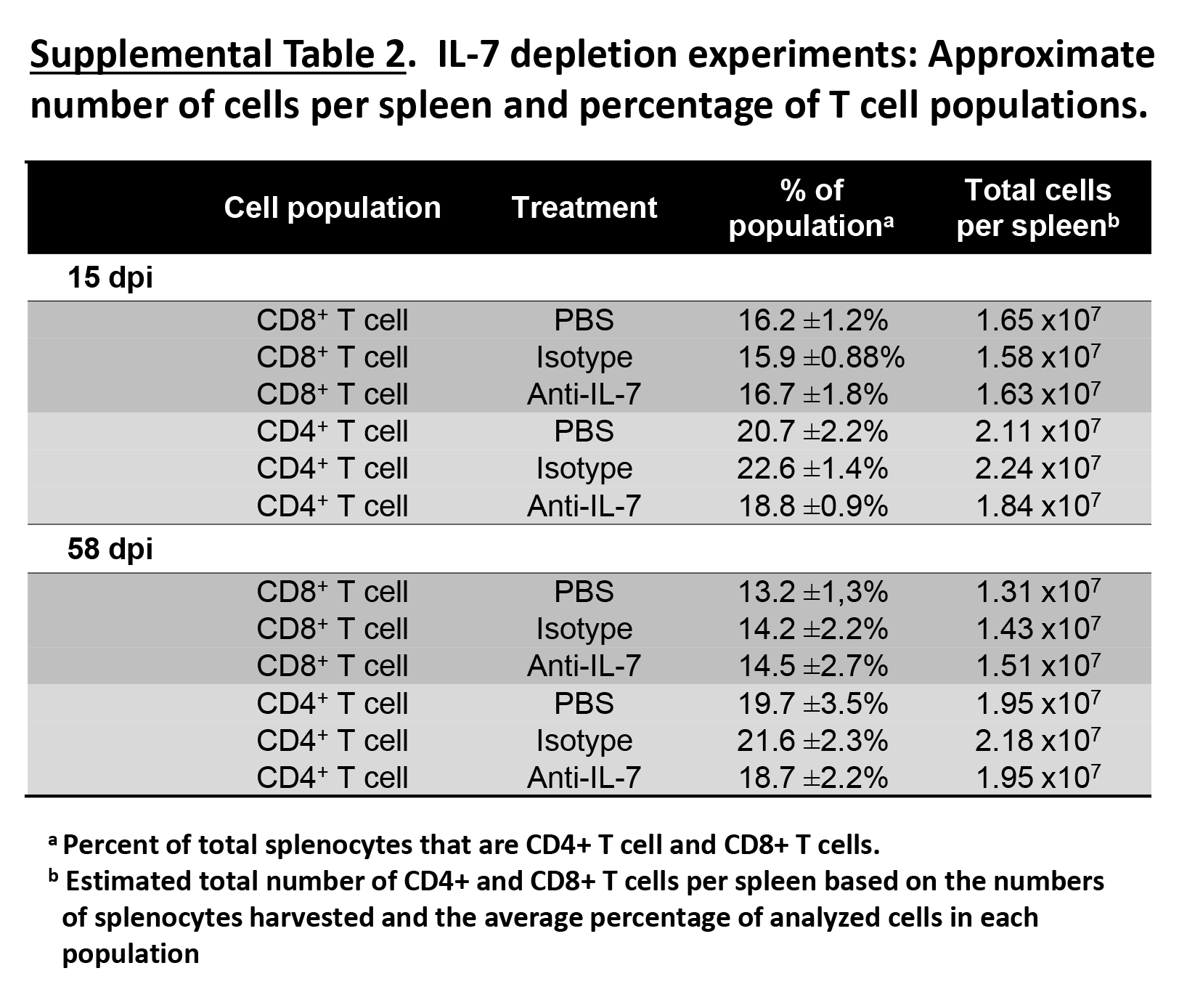

Supplement: Table S2 — Percentage and approximate number of T cell populations per spleen for IL-7 depletion experiments. Percent and approximate number of total splenocytes that were CD4+ and CD8+ T cells. For 15 dpi, n = 3. For 58 dpi, n = 5. (TIF) [file ppat.1003916.s007.tif]

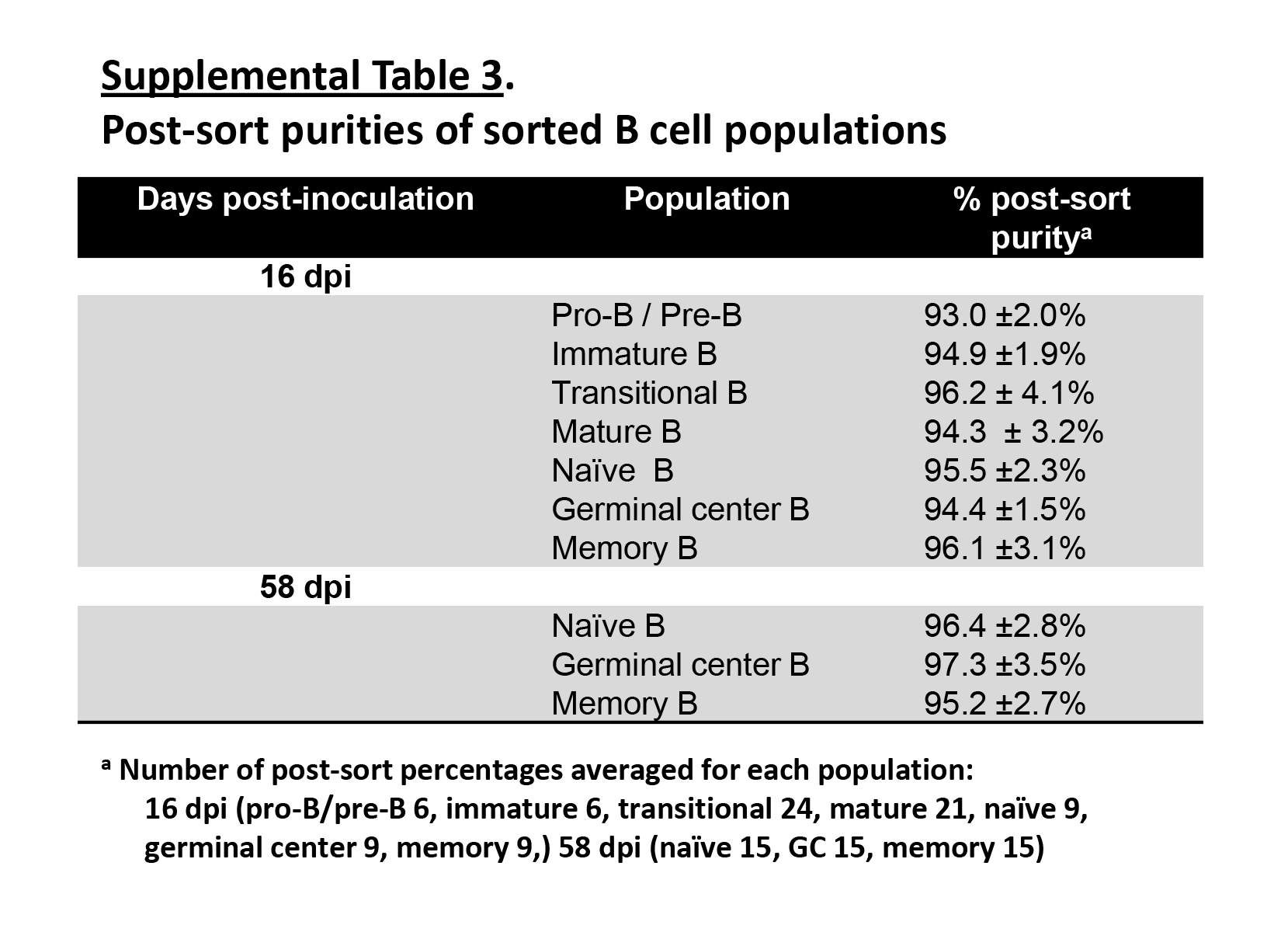

Supplement: Table S3 — Post-sort purities of sorted B cell populations. Post-sort percentages averaged for each population at 16 dpi and 58 dpi. (TIF) [file ppat.1003916.s008.tif]
